# Rapid local and systemic jasmonate signalling drives the initiation and establishment of plant systemic immunity

**DOI:** 10.1038/s41477-025-02178-4

**Published:** 2026-01-06

**Authors:** Trupti Gaikwad, Susan Breen, Emily Breeze, Erin Stroud, Rana Hussain, Satish Kulasekaran, Nestoras Kargios, Fay Bennett, Marta de Torres-Zabala, David Horsell, Lorenzo Frigerio, Pradeep Kachroo, Murray Grant

**Affiliations:** 1 School of Life Sciences, University of Warwick, Coventry, UK; 2 Biopharm Discovery, GlaxoSmithKline, Stevenage, UK; 3 Biosciences, College of Life and Environmental Sciences, University of Exeter, Exeter, UK; 4 College of Engineering, Maths & Physical Sciences, University of Exeter, Exeter, UK; 5 Department of Plant Pathology, University of Kentucky, Lexington, KY USA; 6 Present Address: Marine Biology Association, Plymouth, UK; 7 Present Address: Department of Cell and Molecular Sciences, James Hutton Institute, Invergowrie, UK

**Keywords:** Plant immunity, Plant cell biology

## Abstract

Successful recognition of pathogen effectors by plant disease resistance proteins, or effector-triggered immunity (ETI), contains the invading pathogen through localized hypersensitive cell death. ETI also activates long-range signalling to establish broad-spectrum systemic acquired resistance (SAR). Here we describe a sensitive luciferase (LUC) reporter that captures the spatial–temporal dynamics of SAR signal generation, propagation and establishment in systemic responding leaves following ETI. *JASMONATE-INDUCED SYSTEMIC SIGNAL 1* (*JISS1*) encodes an endoplasmic-reticulum-localized protein of unknown function. *JISS1*::*LUC* captured very early ETI-elicited SAR signalling, which surprisingly was not affected by classical SAR mutants but was dependent on calcium and was also wound responsive. Both jasmonate biosynthesis and perception mutants abolished *JISS1*::*LUC* signalling and SAR to *Pseudomonas syringae*. Furthermore, we discovered that ETI initiated jasmonate-dependent systemic surface electrical potentials. These surface potentials were dependent on both glutamate receptors and JISS1, despite neither *JISS1* loss-of-function nor glutamate receptor mutants altering SAR to *Pseudomonas syringae*. We thus demonstrate that jasmonate signalling, usually associated with antagonism of defence against biotrophs, is crucial to the rapid initiation and establishment of SAR systemic defence responses (including the activation of systemic surface potentials) and that *JISS1*::*LUC* serves as a reporter to further dissect these pathways.

## Abstract (web summary)

A novel reporter captured spatial temporal dynamics of effector-triggered-immunity (ETI)-induced systemic immunity, revealing that signal propagation and establishment in systemic acquired resistance depend on jasmonates. Furthermore, ETI initiates jasmonate-dependent systemic induced surface electrical potentials.

## Main

Despite the discovery of plant systemic acquired resistance (SAR) over a century ago, our knowledge of the signalling processes underlying the establishment, propagation and especially initiation of this response remains fragmentary. Classically, SAR is established following effector-triggered immunity (ETI) leading to the hypersensitive response (HR). SAR has also been reported to be activated via pathogen-associated molecular pattern recognition and virulent bacterial phytopathogens, although the latter has also been reported to trigger systemic induced susceptibility^1,2^.

Multiple molecules are implicated in SAR induction, including salicylic acid (SA) and its volatile derivative methyl salicylate, azelaic acid (AZA), glycerol-3-phosphate, dehydroabietinal, pipecolic acid (Pip) and *N*-hydroxy-pipecolic acid (NHP). More recently, extracellular NAD(P), the volatile monoterpenes α- and β-pinene, vitamin B6 and small RNAs derived from *TAS3a* were shown to induce SAR^3–6^. HR-generated reactive oxygen species (ROS) and nitric oxide (NO) are integral to ETI-initiated SAR, most likely via C_18_ unsaturated fatty acid oxidation of chloroplast lipids^2,7^. Hydrolysis of C_18_ fatty acids released from thylakoid membrane monogalactosyldiacylglycerol and digalactosyldiacylglycerol generates AZA^8,9^. The importance of lipid signalling in SAR is highlighted by the involvement of lipid transfer proteins, AZELAIC ACID INDUCED1 (AZI1) and DEFECTIVE IN INDUCED RESISTANCE1 (DIR1)^8^. Plants defective in SA, glycerol-3-phosphate, NO or ROS biosynthesis have reduced levels of Pip in distal tissues, reinforcing the complex metabolic interplay in the establishment of SAR^10^. Airborne defence cues also activate SAR^11^; thus, one can conclude that multiple signals translocating apoplastically, symplastically^12,13^ and as volatiles can collectively confer broad-spectrum systemic resistance against diverse pathogens, including viral, bacterial, oomycete, fungal and insect pests^2^. The synthesis, activities and interactions of these SAR inducers have been extensively reviewed^10,14–16^.

Despite progress in understanding the individual signalling networks leading to SAR, the spatial–temporal dynamics and interactions of various chemical signals in the SAR pathway remain unclear. Recognition of *Pseudomonas syringae* pv. *tomato* DC3000 (DC) carrying *avrRpm1* (DC*avrRpm1*)^17^ by the Resistance to *P. maculicola 1* (RPM1) disease resistance protein provides a robust ETI model to dissect signal generation and transduction dynamics underlying SAR. We previously demonstrated that RPM1 activation triggers early increases in cytosolic calcium, beginning ~1.5–2 h post-infection (hpi)^18,19^, followed by lipid-peroxidation-triggered biophoton generation ~3 hpi^20,21^ and visible leaf collapse ~6 hpi. RPM1 activation elicits rapid transcriptional reprogramming 4 hpi in systemic leaves, which strongly overlaps with jasmonate-triggered systemic wound responses^22^. Here we report *JASMONATE-INDUCED SYSTEMIC SIGNAL 1* (*JISS1*), a jasmonate-responsive SAR reporter that captures unexpectedly rapid temporal–spatial dynamics following ETI. We show that SAR requires enzymatic production of a local jasmonate signal that propagates via the vasculature and epidermal cells to systemic leaves and is coupled to calcium- and jasmonate-dependent systemic surface electrical potentials.

## Results

### *JISS1* expression reveals temporal and spatial dynamics of early effector–resistance gene interactions

**Fig. 1:**
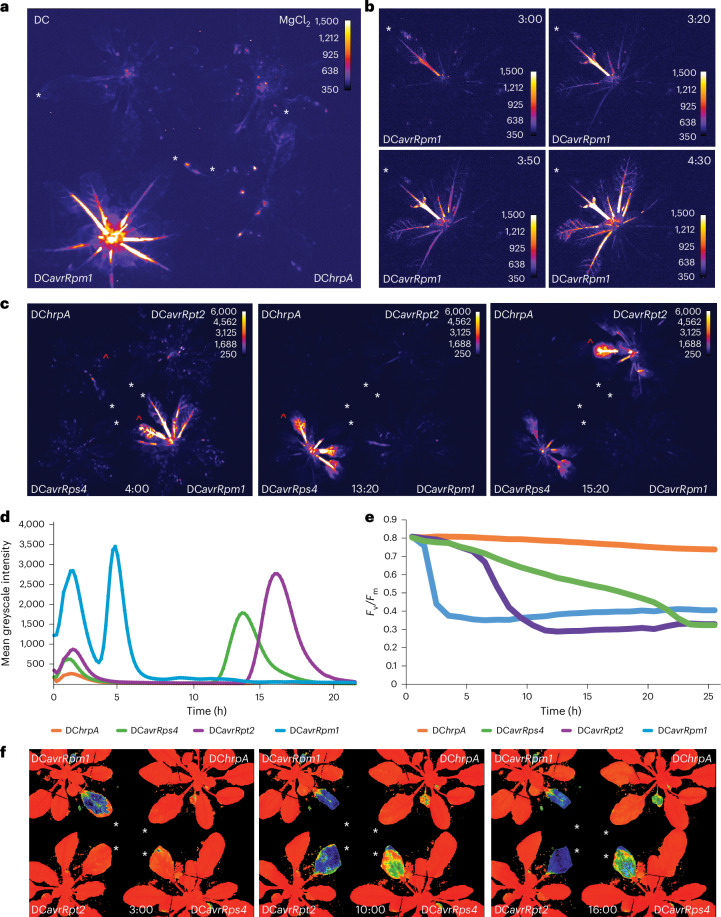
*JISS1* expression is induced systemically by ETI. **a**, Luciferase activity in *JISS1*::*LUC* plants following DC*avrRpm1*, DC, DC*hrpA* or mock (MgCl_2_) challenges at 4:30 hpi. Throughout the figure, white asterisks indicate infiltrated leaves, and red carets indicate leaves used for signal intensity analysis (**d**). The images are false-coloured by signal intensity, as indicated by individual calibration bars. **b**, Temporal spatial dynamics of luciferase activity in *JISS1*::*LUC* plants following DC*avrRpm1* challenge, initiating at 3 hpi. The 3:20 hpi, 3:50 hpi and 4:30 hpi images capture the systemic spread of the signal over time. **c**, Different *Avr* genes display temporal specificity in the activation of systemic *JISS1*::*LUC*: DC*avrRpm1* (4 hpi), DC*avrRps4* (13:20 hpi) and DC*avrRpt2* (15:20 hpi), compared with the DC*hrpA* control. **d**, *JISS1*::*LUC* signal intensity in leaves adjacent to infiltration (red carets in **c**) plotted over time (h). **e**, *F*_v_/*F*_m_ is strongly suppressed during ETI following DC*avrRpm1* (3 hpi), DC*avrRpt2* (10:00 hpi) or DC*avrRps4* (16:00 hpi) challenge compared with DC*hrpA*. **f**, Visualization of *F*_v_/*F*_m_ suppression wherein orange indicates *F*_v_/*F*_m_ of a healthy leaf (~0.8), green represents a reduction in *F*_v_/*F*_m_ as ETI progresses (~0.6) and blue represents a strong impact of ETI on *F*_v_/*F*_m_ (~0.3). Representative images of over ten repeats are shown. Source data

**Extended Data Fig. 1:**
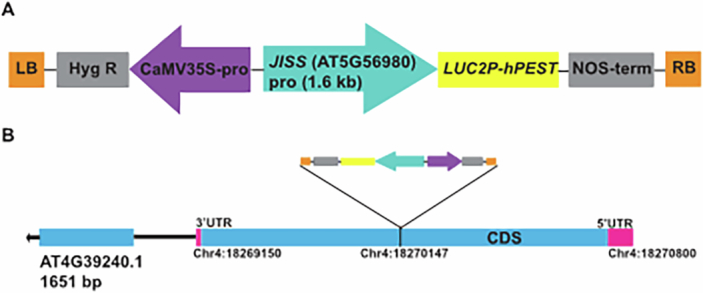
Schematic of the *JISS1:LUC* construct and its T-DNA integration location in the *Arabidopsis* genome. (**A**). The *Photinus pyralis LUC2P* reporter gene (from Promega pGL4-11) was PCR amplified and cloned into pCAMBIA1302 digested with Kpn1 and Pml1 to generate pC1LUCP with a *NOS* terminator. A 1651 bp *JISS1* fragment containing the promoter and encoding the first 84aa of JISS1 was PCR amplified as described^64^ generating a hygromycin selectable *JISS1:LUC* construct. (**B**) Eight independent homozygous lines *A. thaliana* Col-5 *JISS1:LUC* expressing transgenic lines were generated and tested for luciferase expression in systemic leaves following an immunising challenge with DC*avrRpm1*. A homozygous line showing strong *JISS1:LUC* expression was chosen and the genomic location of the *JISS1:LUC* T-DNA construct was determined at position 18270147 in the coding region of AT4G39240.1 sequence analysis as per^70^.

JISS1 (At5g56980; previously known as A70 (ref. ^22^)), a protein of unknown function, is an early SAR marker^22^. To monitor SAR transcriptional dynamics, we fused the promoter of *JISS1* and the sequence encoding the first 84 amino acids of JISS1 to luciferase (Extended Data Fig. 1). Homozygous *JISS1* promoter::*luciferase* (*JISS1*::*LUC*) lines showed rapid systemic luciferase activity following challenge with DC*avrRpm1*, but not with virulent DC; the type-III-secretion-system-deficient DC*hrpA*, which elicits pathogen-associated-molecular-pattern-triggered immunity (PTI) responses; or mock challenge (MgCl_2_) (Fig. 1a). SAR signal propagation was remarkably rapid, with strong luciferase activity first evident in the petiole of the challenged leaf ~3 hpi (Fig. 1b), and within 30 min *JISS1*::*LUC* activity was established^23,24^. This activity spread to adjacent leaves (~4 hpi, Fig. 1b), reaching maximal intensity ~4.5 hpi, ~1 h prior to any visible collapse of the challenged leaf.

**Extended Data Fig. 2:**
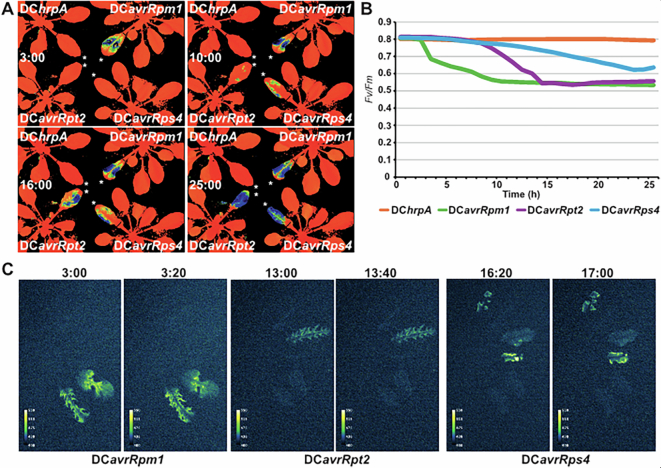
DC*avrRpt2* F_v_/F_m_ dynamics and biophoton generation is earlier than DC*avrRps4* although its systemic *JISS1:LUC* expression is considerably later. (**A, B**) Visualisation (**A**) and quantitation (**B**) of F_v_/F_m_ suppression within a plant over infection time showing, like with biophoton generation (**C**), ETI elicited by DC*avrRpt2* affects chloroplast physiology (PSII) faster than DC*avrRps4* challenge. In (**A**) orange represents a healthy PSII signature whereas blue signifies strongly disrupted PSII due to ETI induced ROS. (**C**) The sequence of biophoton generation, matches F_v_/F_m_ suppression, during ETI elicited by DC*avrRpm1*, DC*avrRpt2* or DC*avrRps4* challenge (times indicated). Stills taken from Supplementary Video 2.

Challenge with DC*avrRpt2* or DC*avrRps4* also induced systemic luciferase activity following recognition by Resistance to *P. syringae* 2 (RPS2)^25^ and RPS4 (ref. ^26^), respectively (Fig. 1c). The spatial pattern of systemic luciferase reporter activity was identical for all ETI responses, but initiation timing differed for each resistance (R) protein, consistent with rapid translocation of an ETI-induced signal (Fig. 1c,d and Supplementary Video 1). To understand the temporal context of R protein elicitation, we investigated local ETI dynamics using two non-destructive physiological readouts, chlorophyll fluorescence and biophoton generation. The chloroplast senses and responds to biotic stress, best exemplified by decreases in the quantum efficiency of photosystem II (*F*_v_/*F*_m_) following ETI elicitation^27^. Quantitative and spatial *F*_v_/*F*_m_ parameters were determined following challenge with DC*avrRpm1*, DC*avrRpt2*, DC*avrRps4* or the DC*hrpA* control (Fig. 1e,f and Extended Data Fig. 2a,b). Biophotons, generated from chloroplast lipid peroxidation^21^ and associated with the initiation of the HR^20^, were additionally assayed (Extended Data Fig. 2c and Supplementary Video 2). Systemic signal initiation elicited by these three R proteins is preceded in the local challenged leaf initially by strong suppression of *F*_v_/*F*_m_ and subsequently by biophoton generation. Although DC*avrRpt2* challenge led to earlier suppression of *F*_v_/*F*_m_ and biophoton generation than DC*avrRps4*, *JISS1*::*LUC* activation was faster following DC*avrRps4* challenge. These data imply that SAR signal generation is not definitively linked to biophoton generation and *F*_v_/*F*_m_ suppression.

### Jasmonates are involved in ETI-induced systemic signalling

**Fig. 2:**
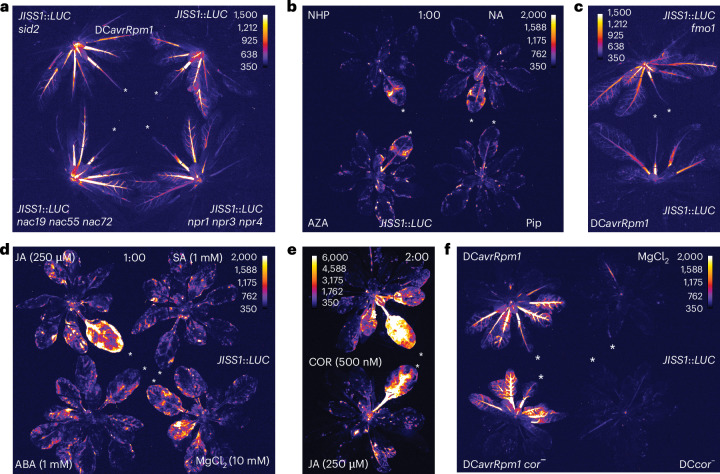
*JISS1*::*LUC* is activated by the jasmonate signalling pathway but not classical SAR elicitors. **a**, The classical SAR mutants *sid2*, *nac19* *nac55* *nac72* and *npr1* *npr3* *npr4* do not alter *JISS1*::*LUC* luciferase signatures following DC*avrRpm1* challenge (4 hpi). Throughout the figure, white asterisks indicate infiltrated leaves, and the images are false-coloured by signal intensity, as indicated by individual calibration bars. **b**, Infiltration of 1 mM Pip, NHP, NA or AZA into leaves of *JISS1*::*LUC* plants does not activate luciferase signal (1 hpi). **c**, No attenuation of luciferase activity is observed in a *JISS1*::*LUC* *fmo1* mutant line following DC*avrRpm1* challenge (4:30 hpi). **d**, JA (250 µM), but not SA (1 mM), ABA (1 mM) or mock (10 mM MgCl_2_) challenge induces local *JISS1*::*LUC* signal propagation (1 hpi). **e**, Luciferase activity in *JISS1*::*LUC* leaves infiltrated with JA (250 µM) or COR (500 nM) (2 hpi). **f**, *JISS1*::*LUC* activity following challenge with DC or the COR-deficient DC mutant DB4G3 (*cor*^−^) with or without *avrRpm1*. DC*avrRpm1* *cor*^−^ induced comparable *JISS1*::*LUC* systemic activity to DC*avrRpm1*, whereas no luciferase activity was detected in DC*cor*^−^ challenged leaves (3:50 hpi). Representative images of at least three repeats are shown.

**Extended Data Fig. 3:**
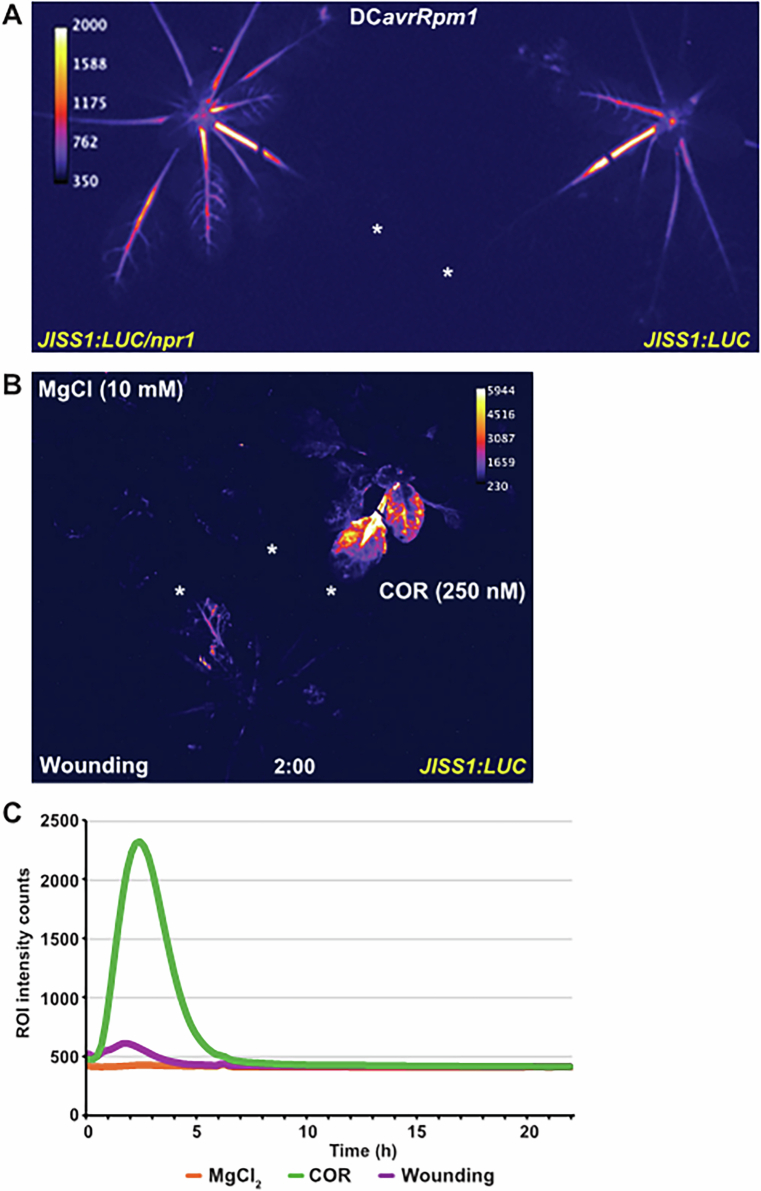
*JISS1* signal propagation is not impaired in *npr1*, but responds to wounding and the JA elicitor coronatine. A white asterisk denotes treated leaves and images are false coloured by signal intensity, indicated by the calibration bar. (**A**) A *JISS1:LUC/npr1* line exhibited comparable systemic luciferase activity to the wild-type Col-0 *JISS1:LUC* line following DC*avrRpm1* challenge (4:20 hpi). (**B**, **C**) Strong and transient luciferase activity was observed in response to 250 nM coronatine (COR), a milder response was triggered by wounding and no response was detected following mock treatment (10 mM MgCl_2_) 2 hpi.

To examine the regulation of *JISS1* in SAR, we crossed the *JISS1*::*LUC* reporter into classical SAR mutant lines expediated by identifying the *JISS1*::*LUC* transfer DNA (T-DNA) insertion position (Extended Data Fig. 1b): *npr1*, where *NONEXPRESSOR OF PATHOGENESIS-RELATED 1* (*NPR1*) encodes a repressor of ETI but is important for SAR^28–30^; *npr1* *npr3* *npr4* (ref. ^28^), impaired in SA signal transduction (notably, NPR3/4 is required for full RPS2 ETI)^31^; *nac19* *nac55* *nac72*, altered in the regulation of SA accumulation^32^; and *SA INDUCTION DEFICIENT 2* (*sid2*), deficient in the accumulation of SA^33^. Surprisingly, when challenged with DC*avrRpm1*, all SAR-compromised lines showed wild-type reporter dynamics (Fig. 2a and Extended Data Fig. 3a). Furthermore, SAR elicitors, AZA (or nonanoic acid (NA), its precursor), Pip and NHP did not significantly increase local JISS1–LUC activity (Fig. 2b), and JISS1–LUC activity in the NHP biosynthetic *FLAVIN-DEPENDENT MONOOXYGENASE 1* (*fmo1*) mutant^34^ was wild-type-like in response to DC*avrRpm1* (Fig. 2c). Together, these data indicate that the signal inducing *JISS1* occurs upstream of or in parallel to previously characterized SAR elicitors.

We next tested key immunity-associated phytohormones for JISS1 induction. Even at high concentrations, leaves infiltrated with SA (1 mM) or abscisic acid (ABA; 1 mM) failed to elicit JISS1–LUC activity. However, jasmonic acid (JA; 250 µM), a key elicitor of systemic wound signalling, induced luciferase locally (Fig. 2d). Collectively, jasmonates comprise JA and its derivatives, with bioactive jasmonoyl-isoleucine (JA-Ile) binding the Skp/Cullin/F-box SCF^COI1^ (CORONATINE-INSENSITIVE PROTEIN 1)–JAZ1 E3 ubiquitin ligase jasmonate co-receptor complex^35,36^. DC does produce the highly active JA-Ile mimic coronatine (COR), but not until ~10 hpi^36,37^. Strikingly, 500 nM COR strongly induced JISS1–LUC locally within 2 h (relative to MgCl_2_ or wounding; Fig. 2e and Extended Data Fig. 3b,c). JISS1–LUC was also induced in plants inoculated with both DC and the DC*cor*^−^ mutant^38^ bacteria expressing *avrRpm1* (Fig. 2f), excluding COR as the systemic elicitor.

### ETI elicits a rapid and propagative jasmonate-dependent signal essential for effective SAR

**Fig. 3:**
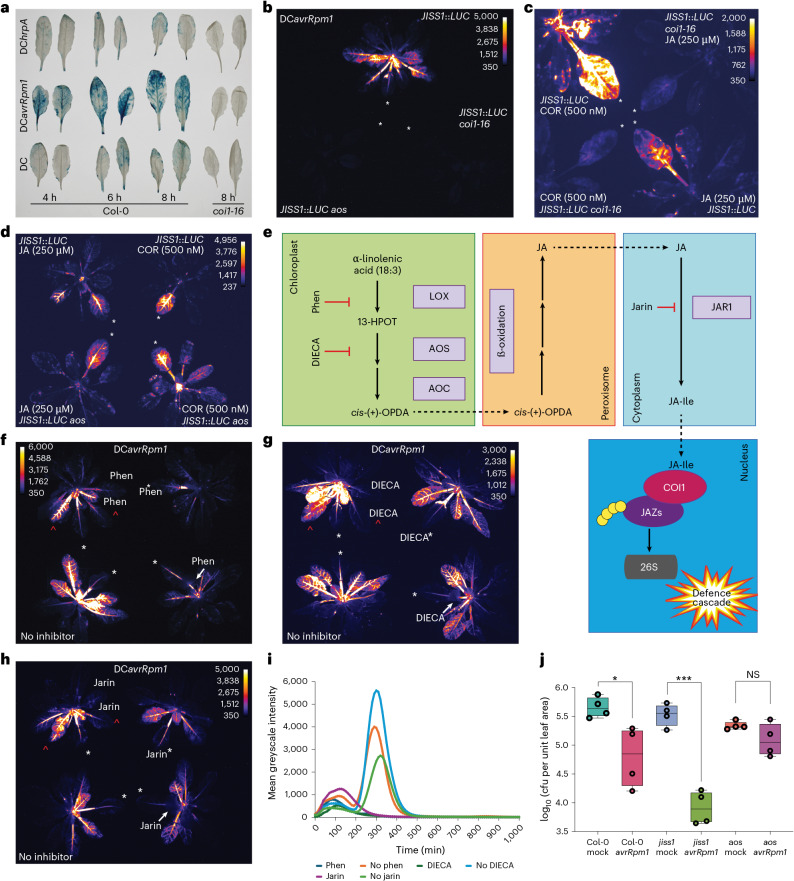
*JISS1*::*LUC* signal propagation is dependent on JA biosynthesis and perception. **a**, DC*avrRpm1* GUS activity in representative systemic leaves of a *JAZ10*::*GUS* reporter line at the times indicated compared to a *JAZ10*::*GUS* *coi1-16* line 8 hpi (*n* = 4 biological replicates). Throughout the figure, white asterisks indicate infiltrated leaves, and red carets indicate leaves used for signal intensity analysis. The images are false-coloured by signal intensity, as indicated by individual calibration bars. **b**, Luciferase activity is not induced in DC*avrRpm1-*treated *JISS1*::*LUC* *aos* or *JISS1*::*LUC* *coi1-16* mutant lines (5 hpi). **c**, Luciferase activity is absent in a *JISS1*::*LUC* *coi1-16* mutant but not *JISS1*::*LUC* leaves 1 hpi following infiltration with 250 µM JA or 500 nM COR (a JA-Ile mimic). **d**, Luciferase activity is restored in *JISS1*::*LUC* *aos* treated with 250 µM JA or 500 nM COR 1 hpi. **e**, Schematic of the JA biosynthetic pathway highlighting the positions of jasmonate inhibitor activity. 13-HPOT, 13-hydroperoxy-9,11,15-octadecatrienoic acid. **f**–**h**, Treatment with phenidone (Phen) (2 mM; 4:40 hpi) (**f**), DIECA (2.5 mM; 5:30 hpi) (**g**) or jarin-1 (25 µM; 5:10 hpi) (**h**) inhibits DC*avrRpm1*-induced *JISS1*::*LUC* activity. In **f**–**h**, the top left image shows the inhibitor pre-infiltrated into the right-hand systemic leaf immediately adjacent to the local challenged leaf, the top right image shows the challenged leaf co-infiltrated with the inhibitor, the bottom right image shows the petiole of the immunized leaf treated with the inhibitor and the bottom left image shows the leaves with no inhibitor. **i**, *JISS1*::*LUC* signal intensity in two leaves adjacent to DC*avrRpm1* infiltration, one treated with inhibitor and one without inhibitor (red carets in **f**–**h**) plotted over time (min). **j**, SAR growth curve of DC following DC*avrRpm1* or mock immunizing challenge on Col-0 (*P = 0.0219, *t* = 3.068, d.f. = 6 (unpaired two-tailed *t*-test)), *jiss1* (***P < 0.0001, *t* = 9.162, d.f. = 6) and *aos* (not significant (NS), *P* = 0.1415, *t* = 1.692, d.f. = 6). (*n* = 4 biological replicates). Box plots represent the minimum, maximum and median values for the statistical analysis of each treatment. Col-0 mock: min = 5.47, max = 5.88, median = 5.64, Q1 = 5.53, Q3 = 5.76; Col-0 DC*avrRpm1*: min = 4.20, max = 5.29, median = 4.85, Q1 = 4.43, Q3 = 5.22; *jiss1* mock: min = 5.26, max = 5.73, median = 5.55, Q1 = 5.45, Q3 = 5.63; *jiss1* DC*avrRpm1*: min = 3.64 max = 4.22, median = 3.89, Q1 = 3.67, Q3 = 4.13; *aos* mock: min = 5.28, max = 5.44, median = 5.32, Q1 = 5.31, Q3 = 5.35; *aos* DC*avrRpm1*: min = 4.81, max = 5.45, median = 5.05, Q1 = 4.88, Q3 = 5.26. The images are representative of at least three repeats.

**Extended Data Fig. 4:**
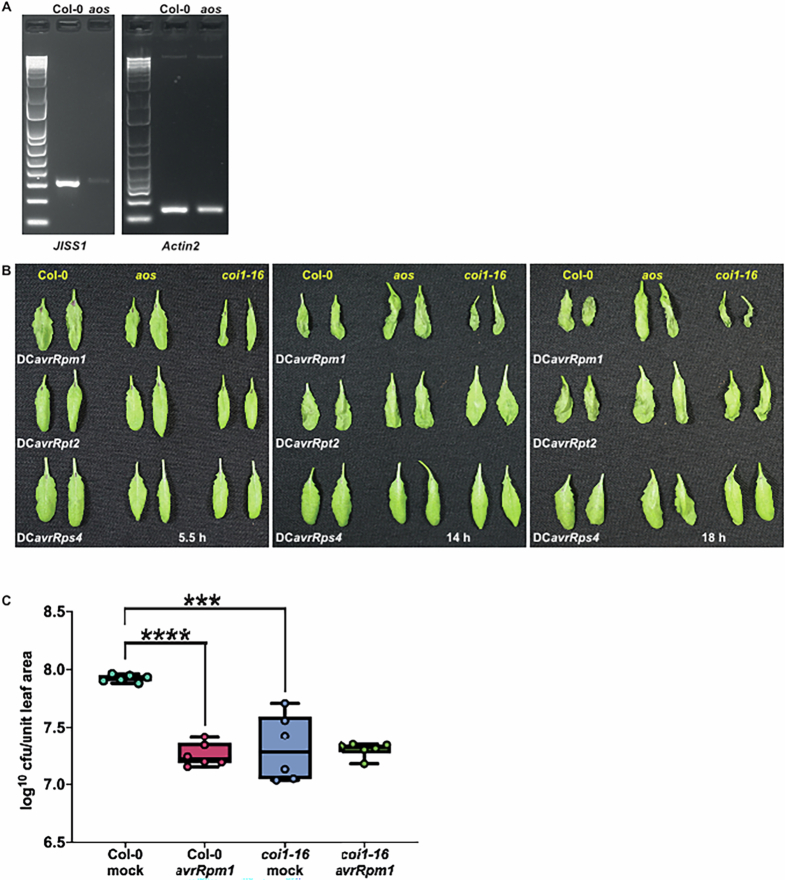
HR development is not affected in mutants with impaired JA biosynthesis (*aos*) or perception (*coi1-16*) but *coi1-16* has reduced disease susceptibility. (**A**) RT-PCR verification loss of *JISS1* expression in the *aos* T-DNA insertion line. *Actin2* expression was used as a loading control (**B**) Phenotyping of Col-0 and *aos* and *coi1-16* mutants showing that loss of JA biosynthesis and signalling does not affect HR development. (**C**) SAR growth curve of *Psm4* following DC*avrRpm1* or mock pre-treatment on Col-0 (**** *p* = <0.0001, t = 15.36, df=10 [unpaired t test]) and the *coi1-16* mutant (*p* = 0.9421, t = 0.07450, df=10). *coi1-16* is more resistant to *Psm4* than wild-type Col-0 (*** p = 0.0004, t = 5.154, df=10) (n = 6 biological replicates). Col-0 mock: min=7.88, max=7.96, median=7.92, Q1 = 7.91, Q3 = 7.94; Col-0 DC*avrRpm1*: min=7.15, max=7.42, median=7.22, Q1 = 7.19, Q3 = 7.31; *coi1-16* mock: min=7.03, max=7.71, median=7.28, Q1 = 7.07, Q3 = 7.53; *coi1-16* DC*avrRpm1*: min=7.19, max=7.36, median=7.33, Q1 = 7.31, Q3 = 7.35. Source data

We reported significant transcriptional overlap between the JA-regulated wounding response and the SAR response at 4 hpi with DC*avrRpm1* compared with mock challenges^22^. Re-examination of these data identified nine *JASMONATE ZIM DOMAIN* (*JAZ*) family members (including *JAZ10*), which act to reimpose the repression of jasmonate signalling^36,39^, induced systemically by DC*avrRpm1* (Supplementary Table 2). As for JISS1, the wound-responsive JAZ10–GUS reporter^40^ is also activated in systemic leaves challenged with DC*avrRpm1*, but not DC or DC*hrpA* (Fig. 3a). This systemic JAZ10–GUS expression is abolished in the jasmonate receptor mutant *coi1-16* (Fig. 3a)^41^. Following DC*avrRpm1* challenge, JISS1–LUC systemic expression was not detected in the JA biosynthetic mutant (*ALLENE OXIDE SYNTHASE*) (*aos*)^42^ (Fig. 3b), and *JISS1* expression was reduced compared with that in Col-0 (Extended Data Fig. 4a). While local application of JA or COR to *JISS1*::*LUC* *coi1-16* failed to elicit local or systemic signals (Fig. 3c), it restored signalling in the *JISS1*::*LUC* *aos* plants (Fig. 3d).

We next determined whether the HR was impacted in *aos* or *coi1-16* mutants. Consistent with biophoton and chlorophyll fluorescence data, collapse of the wild-type Col-0 leaf occurred first with DC*avrRpm1* (~5.5 hpi), followed by DC*avrRpt2* (~14 hpi) and then DC*avrRps4* (~18 hpi). Crucially, timing of the HR was comparable between Col-0 and the *aos* and *coi1-16* mutants (Extended Data Fig. 4b), indicating that the loss of jasmonate biosynthesis or perception does not attenuate local HR but does attenuate JISS1–LUC local and systemic signalling and abolishes SAR. We thus concluded that systemic signalling is both jasmonate and HR dependent.

Aside from JA-Ile, other jasmonate molecules have biological activity in planta^43^, including 12-oxophytodienoic acid (OPDA), although OPDA is not perceived by the SCF^COI1^ receptor complex^44^. We tested the JA biosynthetic inhibitors phenidone, a lipoxygenase inhibitor^45^; diethyldithiocarbamic acid (DIECA), which interferes with octadecanoid signalling^46^; and jarin-1, an inhibitor of JA-Ile synthetase^47^ (Fig. 3e). Concurrent with DC*avrRpm1* challenge, treatment with phenidone (2 mM), DIECA (2.5 mM) or jarin (25 µM), via co-infiltration, petiole application or infiltration into the adjacent systemic responding leaves, abolished or markedly attenuated DC*avrRpm1-*elicited JISS1–LUC activity (Fig. 3f–i). These data independently reinforce a role for jasmonates in SAR, in both local signal generation and establishment in naive responding leaves, and specifically implicate JA-Ile biosynthesis as being necessary for both effective signal transduction and the establishment of SAR.

**Extended Data Fig. 5:**
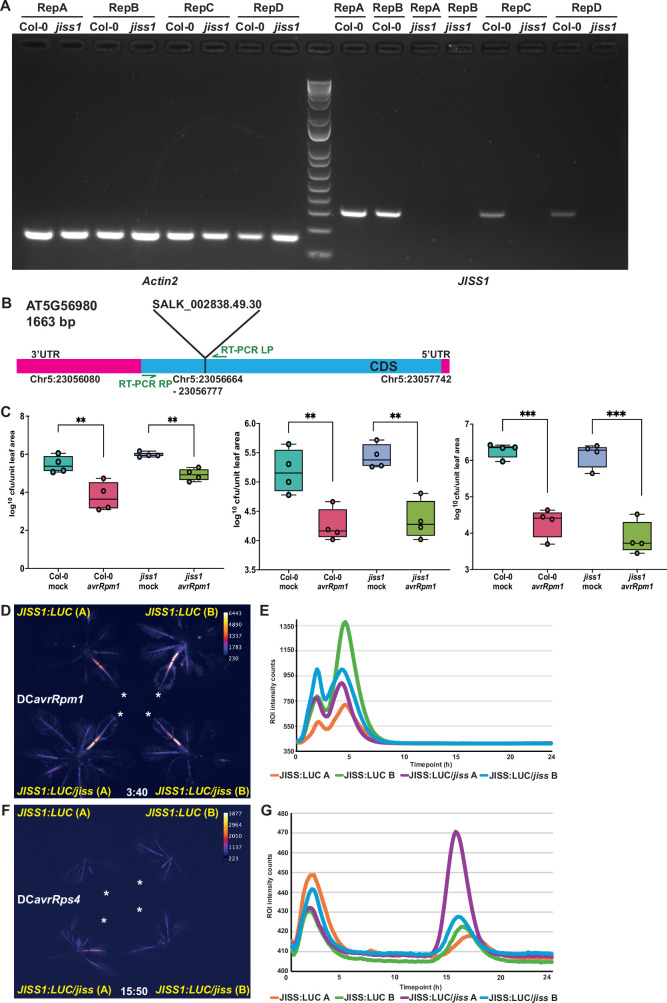
*jiss1* mutants do not impact SAR and the *JISS1* protein is not required for systemic signalling. (**A**) RT-PCR verification loss of *JISS1* expression in the *jiss1* T-DNA insertion line. *Actin2* expression was used as a loading control. **(B)** T-DNA insertion in the coding sequence of the *JISS1* gene (AT5G56980) is located at position Chr5: 23056664–23056777. Relative positions of forward and reverse gene specific RT-PCR primers are indicated, and sequences are given in Methods. **(C)** SAR growth curve of DC following DC*avrRpm1* or mock pre-treatment on wild-type (Col-0) and the *jiss1* mutant (n = 4 biological replicates). **Replicate 1:** Col-0 ** p = 0.0094, t = 3.759, df=6 [unpaired two-tailed t test], *jiss1* ** p = 0.0010, t = 5.943, df=6 [unpaired two-tailed t test]. Col-0 mock: min=5.06, max=6.05, median=5.37, Q1 = 5.12, Q3 = 5.71; Col-0 DC*avrRpm1*: min=3.09, max=4.73, median=3.64, Q1 = 3.18, Q3 = 4.24; *jiss 1* mock: min=5.86, max=6.16, median=5.94, Q1 = 5.92, Q3 = 6.00; *jiss1* DC*avrRpm1*: min=4.56, max=5.31, median=4.92, Q1 = 4.72, Q3 = 5.12. **Replicate 2:** Col-0 ** p = 0.0076, t = 3.947, df=6 [unpaired two-tailed t test], *jiss1* ** p = 0.0015, t = 5.498, df=6 [unpaired two-tailed t test]) Col-0 mock: min=4.78, max=5.65, median=5.15, Q1 = 4.94, Q3 = 5.39; Col-0 DC*avrRpm1*: min=4.02, max=4.66, median=4.16, Q1 = 4.11, Q3 = 4.31; *jiss 1* mock: min=5.26, max=5.72, median=5.38, Q1 = 5.27, Q3 = 5.53; *jiss1* DC*avrRpm1*: min=4.02, max=4.81, median=4.28, Q1 = 4.17, Q3 = 4.45. **Replicate 3:** Col-0 *** p = 0.0001, t = 8.699, df=6 [unpaired two-tailed t test], *jiss1* *** p = 0.0002, t = 7.951, df=6 [unpaired two-tailed t test]. Col-0 mock: min=5.97, max=6.42, median=6.35, Q1 = 6.25, Q3 = 6.37; Col-0 DC*avrRpm1*: min=3.70, max=4.63, median=4.41, Q1 = 4.21, Q3 = 4.49; *jiss 1* mock: min=5.64, max=6.40, median=6.28, Q1 = 6.09, Q3 = 6.34; *jiss1* DC*avrRpm1*: min=3.45, max=4.52, median=3.72, Q1 = 3.65, Q3 = 3.93. **(D-G)** The *JISS1:LUC* reporter in the *jiss1* mutant responds as wild type following challenge with either DC*avrRpm1* (**D**-**E**) or DC*avrRps4* (**F**-**G**). A white asterisk denotes infiltrated leaves and images are false coloured by signal intensity, as indicated by calibration bar. Luciferase activity shows similar dynamics and signal intensity in response to DC*avrRpm1* (**D**-**E**) and DC*avrRps4* (**F**-**G**) in *JISS1:LUC* and *JISS1:LUC/jiss1* lines. Source data

The *coi1* mutant is known to be more resistant to local DC challenge than wild-type *Arabidopsis*^41^. We assessed SAR to different *P. syringae* pathovars (less virulent *P. syringae* pv. *maculicola* race 4 (*Psm4*) or more virulent DC) in the *aos* and *coi1-16* mutants alongside a *JISS1* T-DNA insertion loss-of-function line (*jiss1*; Fig. 3j and Extended Data Figs. 4c and 5a,b). Crucially, neither *coi1-16* nor *aos* exhibited SAR relative to Col-0 across multiple independent assays (*n* > 4). Interestingly, *jiss1* lines still induced SAR following DC*avrRpm1* challenge (Fig. 3j and Extended Data Fig. 5c).

These data collectively imply that ETI elicits rapid de novo synthesis of a jasmonate-dependent signal that propagates systemically and is essential for the effective establishment of SAR. *JISS1* expression is intimately linked to this signal, but JISS1 itself is not critical for SAR or SAR signalling (Extended Data Fig. 5c–f), and its precise biological function remains unclear.

### JISS1 signal localizes to the vasculature and epidermal endoplasmic reticulum

**Fig. 4:**
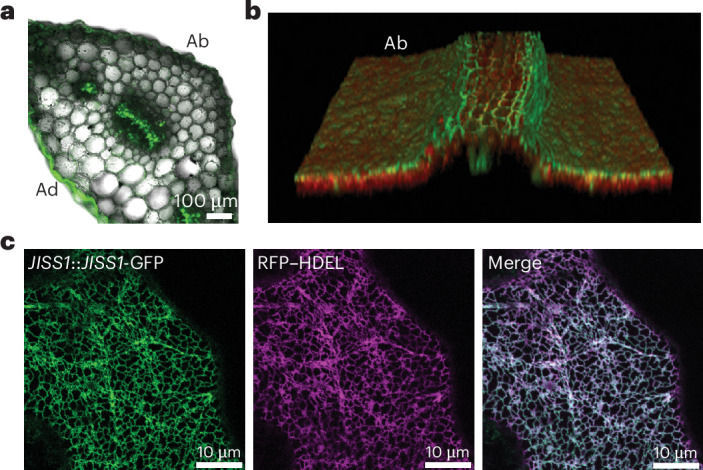
JISS1 signal propagates symplastically through the epidermis and vasculature and localizes to the ER. **a**–**c**, Confocal images of global *JISS1*::*JISS1-GFP* expression in representative systemic leaves following DC*avrRpm1* challenge. Panel **a** shows *JISS1*::*JISS1*^*1–84*^*-GFP* expression in the petiole of a challenged leaf showing JISS1–GFP signal predominantly in the vasculature and epidermal cells. Ab, abaxial surface; Ad, adaxial surface. Scale bar, 100 µm. Panel **b** shows a still from Supplementary Video 3 of JISS1(1–84)–GFP highlighting that the GFP signal is predominately restricted to the central vasculature and abaxial epidermal cell layer. Panel **c** shows representative confocal images of *Arabidopsis* epidermal leaf cells stably expressing *JISS1*::*JISS1-GFP* (green) and the ER luminal marker RFP–HDEL (magenta), which shows that JISS1 localizes strictly to the ER. Scale bars, 10 µm. The images are representative of at least three biological replicates.

**Extended Data Fig. 6:**
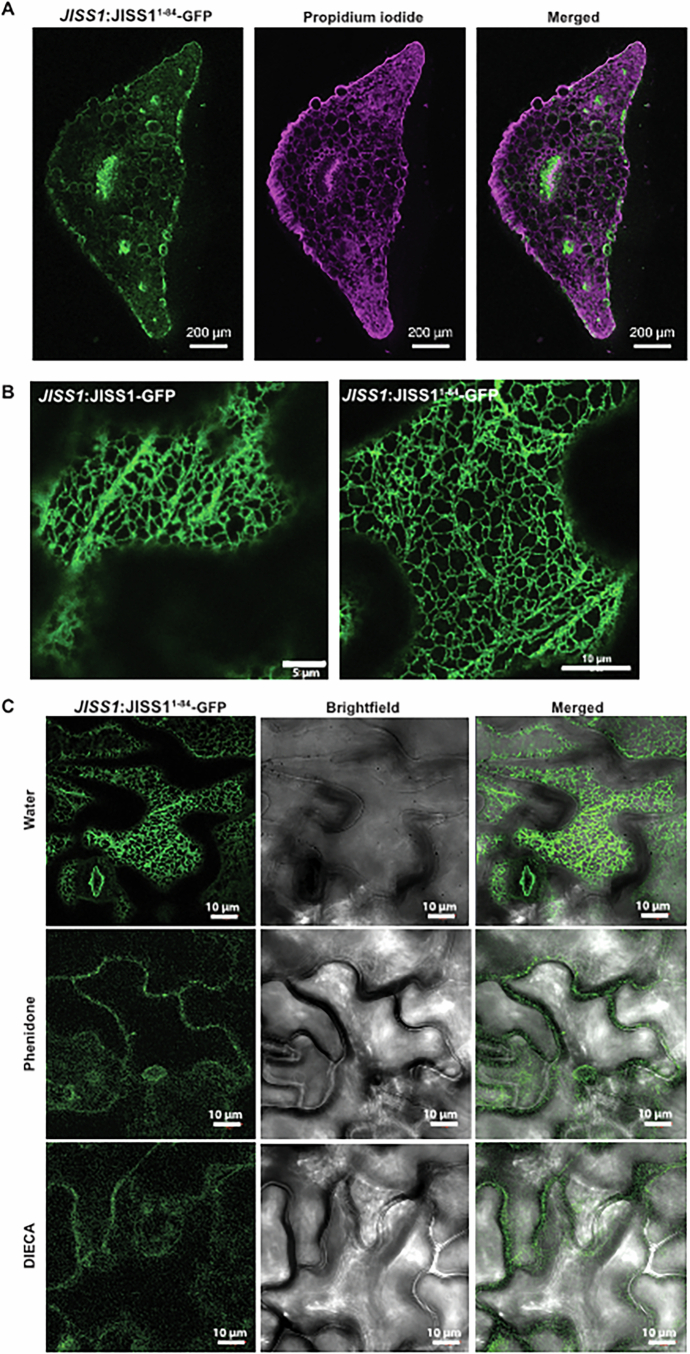
JA dependent localisation of *JISS1:*JISS1^1–84^*-GFP* and *JISS1:*JISS1*-GFP* in systemic tissue. (**A**) Petiole section from systemic leaf showing propidium iodide staining (magenta) and *JISS1:*JISS1^1–84^*-GFP* expression (green) with merged image. Scale bar, 200 µm (**B**) Representative confocal images of *JISS1:*JISS1-GFP and *JISS1:*JISS1^1–84^-GFP plants following DC*avrRpm1* challenge, showing identical subcellular localisation of GFP in ER network of epidermal cells. Scale bars, 5 µm and 10 µm, respectively. **(C)** Representative confocal images of Arabidopsis epidermal leaf cells of *JISS1:*JISS1^1–84^-GFP systemic leaves pre-treated with JA inhibitors, phenidone (2 mM) and DIECA (2.5 mM) prior to DC*avrRpm1* challenge or mock treated. Mock treatment of systemic leaves shows JISS1^1–84^-GFP labelling of the entire ER network 4 hpi whereas Systemic leaves infiltrated with phenidone or DIECA exhibit strongly reduced GFP signal in the ER 4 hpi compared to signal across the entire ER network after mock treatment. Scale bar, 10 µm. Images are representative of at least 3 biological replicates.

We next investigated JISS1 subcellular localization and expression following ETI elicitation. JISS1 was annotated as chloroplast localized (TAIR10; AtSubP). We thus generated both a full-length (*JISS1*_*pro*_::*JISS1-GFP*) and a truncated JISS1–GFP fusion (*JISS1*_*pro*_::*JISS1*^*1–84*^*-GFP*); the latter included the predicted chloroplast transit peptide. In both lines, GFP signal was detected in the systemic leaf within 4.5 hpi, predominantly in both the vasculature and epidermal cells of the petiole and lamina (Fig. 4a,b and Extended Data Fig. 6a). Within the epidermal cells, both versions of the JISS1–GFP fusion proteins surprisingly localized to the endoplasmic reticulum (ER) network, colocalizing with the ER luminal marker, RFP–HDEL (Fig. 4c and Extended Data Fig. 6b). These data are suggestive of a SAR signal moving symplastically and are consistent with the abolition of SAR in plasmodesmata-permeability-restricted *Arabidopsis* overexpressing PLASMODESMATA-LOCATED PROTEIN 5 (ref. ^13^). As observed with JISS1–LUC activity, GFP expression in systemic leaves was markedly suppressed by pretreatment with the JA biosynthetic inhibitors phenidone and DIECA (Extended Data Fig. 6c).

### Systemic electric signal propagation is a general feature of ETI activation

**Fig. 5:**
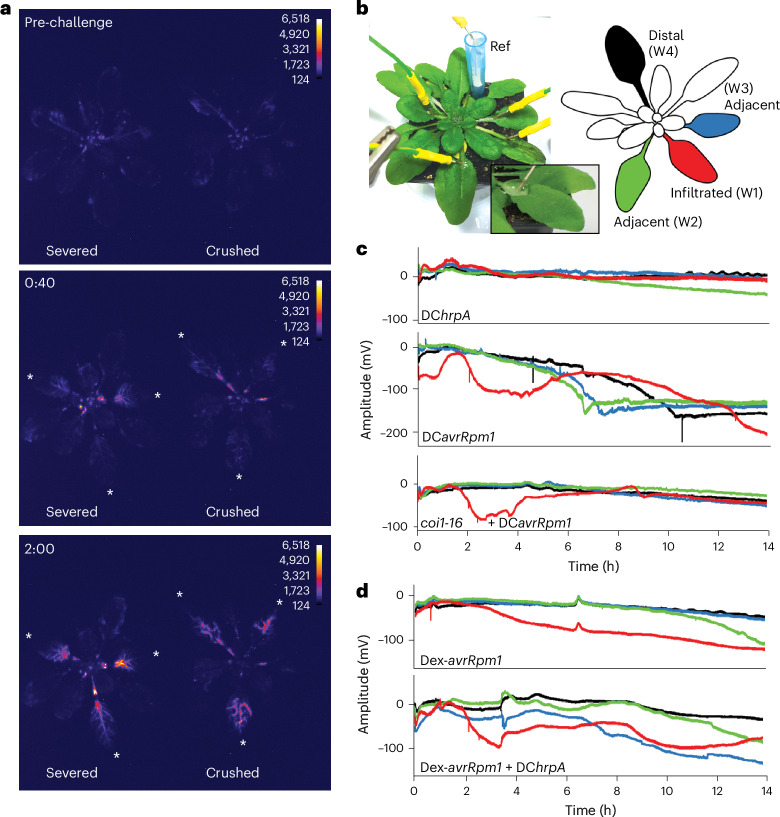
Systemic electric signal propagation is a general feature of ETI activation. **a**, White asterisks indicate wounded *JISS1*::*LUC* leaves, and the images are false-coloured by signal intensity, as indicated by individual calibration bars. Luciferase activity in severed or crushed leaves (*n* = 3) initiated and peaked at ~40 min and ~2 h post-wounding, respectively. **b**, Plant electrophysiology experimental set-up with a cartoon showing spatial sampling and colour coding of leaves for working electrodes (W). The inset illustrates electrode positioning. Ref, reference electrode. **c**, Leaves challenged by DC*avrRpm1* but not DC*hrpA* (red) show an initial depolarization ~2 hpi and subsequent repolarization. From 4 to 7 hpi, SISPs are propagated in the two systemic leaves (blue and green) adjacent to the DC*avrRpm1*-immunized leaf, with the distal leaf (black) responding later (from ~7 hpi). No SISPs are observed in the DC*hrpA* treatments. The *coi1-16* mutant shows depolarization of the DC*avrRpm1*-challenged leaf but no SISP initiation. **d**, Dex-induced *avrRpm1* expression does not replicate DC*avrRpm1*-induced SISPs; however, infiltration of DC*hrpA* 1 h after Dex application re-instigates SISPs. The experiments were repeated at least twice (see Extended Data Fig. 7 for further details).

Wound-induced accumulation of JA and JA-Ile in undamaged distal leaves, together with altered expression of jasmonate-responsive genes, is preceded by the rapid generation of wound-activated surface electrical potentials (WASPs), caused by plasma membrane depolarization^40^. WASPs are mediated by glutamate-triggered systemic activation of cytosolic Ca^2+^ signalling via specific vasculature-localized members of the glutamate-like receptor family, GLR3.3 and GLR3.6 (refs. ^40,48^). These cation-permeable ion channels also contribute to systemic defence against herbivory^48^. To confirm the link between *JISS1* and JA signalling, we assayed the activity of *JISS1*::*LUC* in response to wound stimuli. Either crushing with forceps or severing the leaf at the petiole triggered rapid, transient induction of *JISS1*::*LUC* (Fig. 5a). Wounding induced a much weaker signal than COR application (Extended Data Fig. 3b,c). Given the similar spatial distribution of WASPs and JISS1–LUC signals, and the transcriptional parallels between wounding and early SAR responses^22^, we measured leaf surface potentials between electrodes attached to the midrib/petiole junction of a local challenged leaf (‘infiltrated’) and to fully expanded leaves immediately adjacent to (‘adjacent’) and directly opposite (‘distal’) the challenged leaf (Fig. 5b).

**Extended Data Fig. 7:**
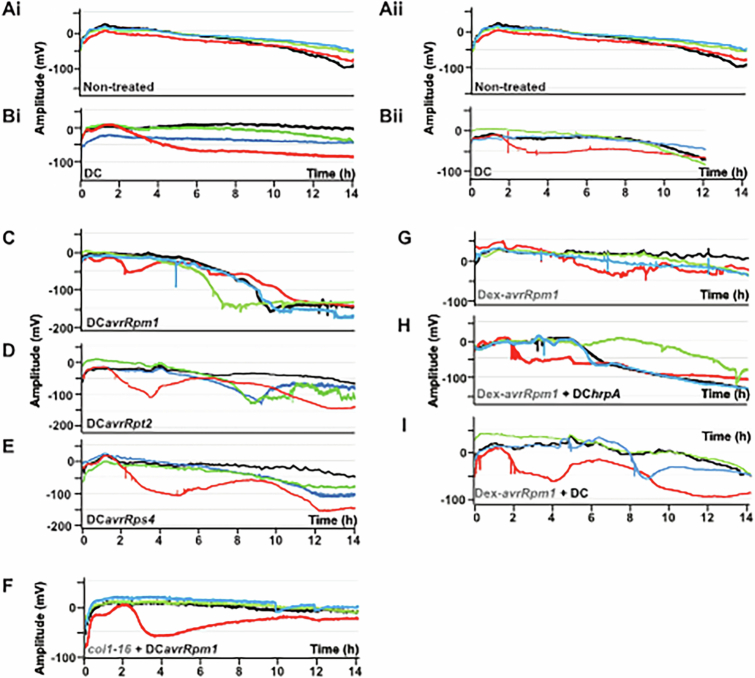
ETI activation of systemic electric signalling is consistent. Replicated data displayed consistent with Fig. 5. (**A**) Col-0 plants do not show development of SISPs in non-treated plants. (**B**) Col-0 plants do not show development of SISPs following DC challenge. (**C-E**) Challenge with DC*avrRpm1*, DC*avrRpt2*, and DC*avrRps4* exhibits depolarisation of the infected leaf from 2 hpi, followed by induction of SISPs from 6-10 hpi. (**F**) Infiltration of DC*avrRpm1* in the *coi1-16* mutant causes transient depolarization (3-4 hpi) in the challenged but not distal nor adjacent leaves. (**G**) Dex-avrRpm1 plants do not show SISPs in non-treated plants. (**H-I**) Infiltration of DC*hrpA* or DC 1 h after Dex induction of *avrRpm1* induces depolarisation and repolarisation of the infiltrated leaf and initiation of SISPs in some systemic responding leaves, albeit weaker than DC*avrRpm1*.

No specific changes in leaf surface potentials were induced in untreated or DC*hrpA*-challenged leaves, whereas DC induced a small depolarization of the local challenged leaf (Fig. 5c and Extended Data Fig. 7a,b). By contrast, challenge with DC*avrRpm1* initially induced strong depolarization with an amplitude of about −100 mV over a duration of ~2 h, followed by repolarization (Fig. 5c and Extended Data Fig. 7c). The timing of depolarization strongly correlated with biophoton generation, the suppression of *F*_v_/*F*_m_ and JISS1–LUC systemic signal initiation (Fig. 1 and Extended Data Fig. 2). Following repolarization of the challenged leaf, systemic immunity surface potentials (SISPs) were detected in adjacent leaves with maximal depolarization ~7 hpi. SISPs were also detected in distal leaves, but maximal depolarization occurred later (~10 hpi; Fig. 5c and Extended Data Fig. 7c). These SISPs, unlike WASPs and herbivory responses, which travel at speeds in excess of millimetres per second^49^, represented slower variation potentials, which largely mirrored the spatial dynamics of systemic JISS1–LUC activity, albeit with delayed propagation. As both DC*avrRpt2* and DC*avrRps4* also trigger SISPs (Extended Data Fig. 7d,e) with the timing of initiation largely consistent with the initiation of suppression of *F*_v_/*F*_m_ (Fig. 1e,f, and Extended Data Fig. 2a,b), we concluded that SISPs are specifically elicited by ETI.

Consistent with jasmonate dependency, SISPs were abolished in *coi1-16*, with only the DC*avrRpm1*-challenged leaf (red trace) undergoing initial depolarization (Fig. 5c and Extended Data Fig. 7f) Surprisingly, despite eliciting a local visible HR, SISPs were not induced by conditional (dexamethasone (Dex)) induction of *avrRpm1*. Instead, a steady depolarization of the induced leaf with no subsequent repolarization was observed (Fig. 5d and Extended Data Fig. 7g). However, co-infiltration of Dex-induced leaves with DC*hrpA* or DC (Fig. 5d and Extended Data Fig. 7h,i) broadly recapitulated SISP changes, implying that PTI is required for SISP generation.

**Fig. 6:**
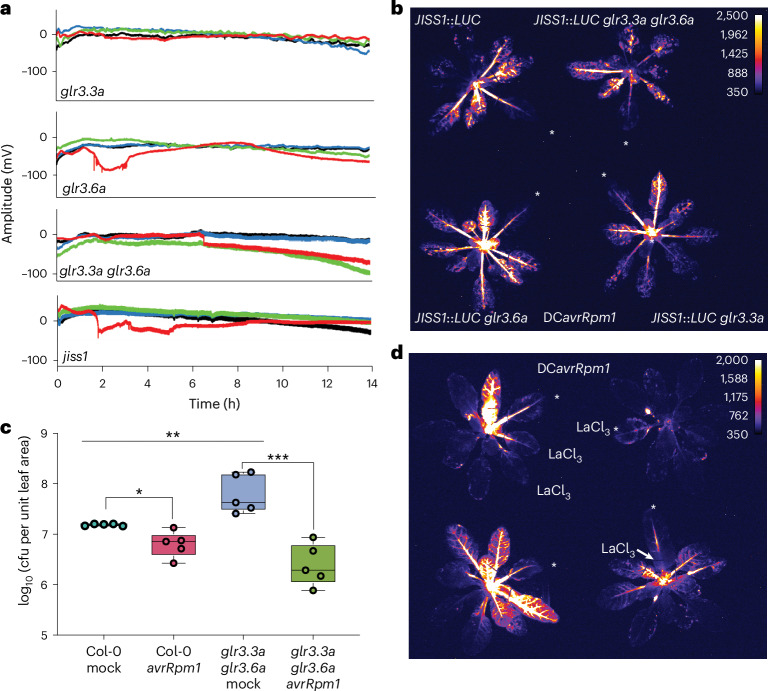
JISS1 systemic signal is calcium dependent. **a**, Glutamate receptor mutants *glr3.3a*, *glr3.6a* and *glr3.3a* *glr3.6a* do not propagate SISPs following DC*avrRpm1* challenge. *glr3.6a*, like *jiss1*, shows limited depolarization of the DC*avrRpm1*-challenged leaf. **b**, DC*avrRpm1*-challenged *JISS1*::*LUC* *glr3.3a* *glr3.6a*, *JISS1*::*LUC* *glr3.3a* and *JISS1*::*LUC* *glr3.6a* mutants all exhibited comparable systemic luciferase activity to that of *JISS1*::*LUC* (4:10 hpi). In **b** and **d**, white asterisks indicate infiltrated leaves, and luciferase images are false-coloured by signal intensity, as indicated by the calibration bars. **c**, SAR growth curve of *Psm4* following DC*avrRpm1* or mock pretreatment on Col-0 (*P = 0.0102, *t* = 3.343, d.f. = 8 (unpaired two-tailed *t*-test)) and the *glr3.3a* *glr3.6a* mutant (***P = 0.0005, *t* = 5.550, d.f. = 8). *glr3.3a glr3.6* mutant mock and the Col-0 mock were significantly different (**P = 0.0076, *t* = 3.593, d.f. = 8) (*n* = 5 biological replicates). Box plots represent the minimum, maximum and median values for the statistical analysis of each treatment. Col-0 mock: min = 7.16, max = 7.20, median = 7.19, Q1 = 7.17, Q3 = 7.20; Col-0 DC*avrRpm1*: min = 6.43, max = 7.13, median = 6.86, Q1 = 6.71, Q3 = 6.88; *glr3.3a* *glr3.6a* mock: min = 7.41, max = 8.23, median = 7.63, Q1 = 7.52, Q3 = 8.18; *glr3.3a* *glr3.6a* DC*avrRpm1*: min = 5.88, max = 6.94, median = 6.29, Q1 = 6.17, Q3 = 6.67. **d**, Treatment with the calcium inhibitor LaCl_3_ (1 mM) inhibits DC*avrRpm1*-induced *JISS1*::*LUC* activity compared with the control (4:50 hpi). Top left, two systemic leaves below the challenged leaf were pre-infiltrated with LaCl_3_. Top right, local leaf co-infiltrated with LaCl_3_ and DC*avrRpm1*. Bottom right, petiole of an immunized leaf treated with LaCl_3_. Bottom left, no inhibitor. All experiments were repeated at least three times with similar results. SAR (**c**) was evident with either DC and *Psm4* challenge or secondary challenges.

**Extended Data Fig. 8:**
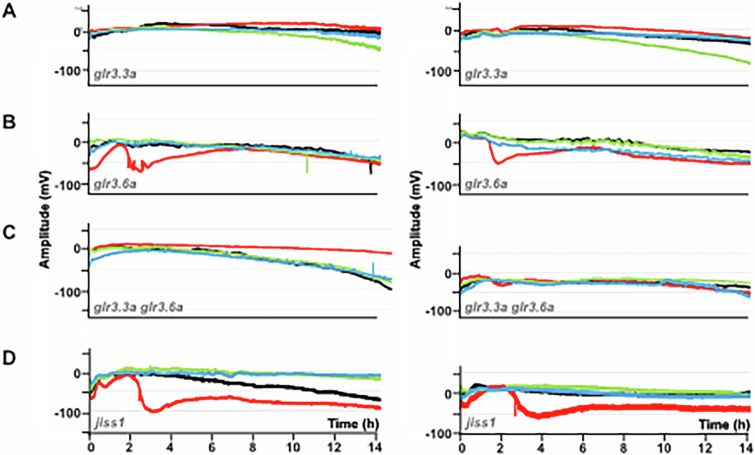
ETI activation of systemic electric signalling is consistent. Replicated data (n = 2) displayed consistent SISP signatures to Fig. 6. (**A**) Challenge of *glr3.3a* with DC*avrRpm1* does not show SISPs. (**B**) Challenge of *glr3.6a* with DC*avrRpm1* shows limited depolarization in the infiltrated but not distal leaves. (**C**) Challenge of *glr3.3a glr3.6a* with DC*avrRpm1* does not show SISPs. (**D**) Challenge of *jiss1* with DC*avrRpm1* shows limited depolarization in the infiltrated but not distal leaves.

We also measured SISPs in *glr3.3a*, *glr3.6a*, *jiss1* and the *glr3.3a* *glr3.6a* mutant, the latter of whose functions are necessary for full wound and herbivory responses^40,50^. Mirroring WASP attenuation^40^, SISPs were abolished in the *glr3.3a*, *glr3.6a* and *glr3.3a* *glr3.6a* mutants (Fig. 6a and Extended Data Fig. 8a–c). *glr3.6a* showed depolarization of the local challenged leaf (Fig. 6a and Extended Data Fig. 8b)^50^, which mirrored *jiss1* and *coi1-16* responses in which SISPs were abolished and depolarization was only seen in the local challenged leaf (Figs. 5c and 6a and Extended Data Figs. 7f and 8d).

### Calcium signalling is necessary for systemic immunity

Given the finding that ETI alone is not sufficient to induce SISPs, we crossed *JISS1*::*LUC* into the *glr3.3a*, *glr3.6a* and *glr3.3a* *glr3.6a* mutants. Interestingly, JISS1–LUC dynamics were unaltered in all loss-of-function *glr* lines tested (Fig. 6b), though both *jiss1* and *glr* mutants lost the ability to generate SISPs.

AvrRpm1–RPM1 mediated SISP generation thus requires functional JISS1, COI1 and vasculature-specific GLR Ca^2+^ channels, which link systemic electrical signal propagation in response to wounding^40^ and herbivory^50^. As the loss of GLRs does not abolish JISS1–LUC activity or SAR to *Psm4* (Fig. 6c), SISPs most likely encode another SAR signal, for example for herbivory responses. Since SISPs, like WASPs, are dependent on GLR3.3, which triggers long-distance calcium signalling^51^, we investigated the role of calcium in JISS1–LUC activity. The calcium channel blocker LaCl_3_ (1 mM) abolished DC*avrRpm1*-induced JISS1–LUC activity in systemic leaves, as did co-infiltration of LaCl_3_ with DC*avrRpm1*. Also, the application of LaCl_3_ to the petiole surface markedly attenuated JISS1–LUC activity (Fig. 6d). Therefore, Ca^2+^ signalling, which is intimately linked to ETI^52^, is necessary for JISS1–LUC SAR activity and SISP propagation.

## Discussion

SAR confers broad-spectrum resistance to viral, fungal, oomycete and bacterial pathogens and insect pests despite their diverse lifestyles and virulence strategies. A variety of signalling molecules have been shown to be important for SAR. Many of these are synthesized de novo, implying upstream inductive signals. Our current understanding of signal generation, translocation and establishment in systemic responding leaves is constrained by a lack of information on the spatial–temporal dynamics of SAR activation. Here we used *JISS1* (previously *A70*)^22^, a jasmonate-responsive gene, to develop a reporter that overcomes these constraints to faithfully report ETI-elicited SAR. Using a *JISS1*::*LUC* reporter, chlorophyll fluorescence^27,53^ and biophoton^20^ imaging, we characterized SAR dynamics during HR elicited by two coiled-coil nucleotide-binding leucine-rich repeats (CNLs,RPM1 and RPS2) and a Toll-like/Interleukin 1 nucleotide-binding leucine-rich repeat receptor (TNL,RPS4). We demonstrate that jasmonate signalling is essential for SAR and further show that the ER-localized JISS1 is essential for the generation of electrical surface potentials in systemic responding leaves (SISPs), like those reported to underpin resistance to herbivory^50^.

*JISS1*::*LUC* signal generation was unaffected in the mutants *sid2*, *npr1* *npr3* *npr4*, *fmo1* and *nac19* *nac55* *nac72* (Fig. 2 and Extended Data Fig. 3a). Infiltration of the SAR inducers NA, AZA, Pip and NHP also failed to induce *JISS1*::*LUC*. Of the classic plant immunity-associated hormones, neither ABA nor, unexpectedly, SA activated *JISS1*::*LUC*. JA and COR elicited local but not systemic luciferase activity, and COR-deficient DC*avrRpm1* still replicated *JISS1*::*LUC* temporal and spatial activation dynamics (Fig. 2).

Consistent with a key role for jasmonates in establishing SAR, both jasmonate biosynthetic (*aos*) and signalling (*coi1-16*) mutants were SAR deficient and failed to activate JISS1–LUC following DC*avrRpm1* challenge (Fig. 3b). These data expand our previous results^22^ demonstrating compromised SAR in the JA biosynthetic mutant *opr3* (*12-OXOPHYTODIENOATE REDUCTASE 3*) and the JA signalling mutant *jin1* (*JASMONATE-INSENSITIVE 1*; *myc2*)^22^. Neither COR nor JA infiltration activated luciferase in *coi1-16* *JISS1*::*LUC* plants (Fig. 3c), but their exogenous application restored activity in *JISS1*::*LUC* *aos* (Fig. 3d). Reinforcing the importance of jasmonate signalling, the JA-Ile synthetase inhibitor jarin (like phenidione and DIECA) significantly attenuated *JISS1*::*LUC* signal generation, propagation and distal activation (Fig. 3f–i). Interestingly, *jiss1* was SAR competent, and *jiss1* *JISS1*::*LUC* lines were activated by ETI, indicative of a mobile jasmonate-dependent SAR signal propagating through the systemic tissue (Fig. 3j and Extended Data Fig. 5c–g). Thus, genetically, pharmacologically and spatially, via real-time transcription monitoring, we conclusively show a key role for jasmonates in SAR, independent of the classical SAR mutant lines *npr1*, *sid2*, *npr1* *npr3* *npr4* and *nac19* *nac55* *nac72*.

It may seem counterintuitive that jasmonates underpin SAR, given well-documented JA/SA antagonism in biotrophic immunity; however, SA/JA synergism has been reported^54^. Spatial separation of JA and SA signalling during ETI would be a parsimonious explanation for SA/JA dependency and reinforce the importance of short-distance local cellular signalling in ETI responses^55^. Indeed, dual-expressed SA and JA reporters exhibit temporally separated distinct concentric domains (inner/early for SA and outer/later for JA) between the ETI-responding cells^56^, elegantly explaining the COI1 dependency of the *JISS1* reporter and the need to consider spatial cellular context during ETI signalling.

Our data support the model that early ETI leads to chloroplast ROS generation, indicative of the strong suppression of *F*_v_/*F*_m_^27^ and subsequent biophoton generation^20,21^. Enzymatic and non-enzymatic chloroplast galactolipid-derived oxylipins^57,58^ are thus potential substrates for the generation of jasmonate-based mobile SAR signals and JISS1–LUC activation. Both fatty acid desaturase and chloroplast galactolipid mutants necessary for JA synthesis are SAR deficient^7,9^, whereas JA levels increase ~75-fold within 5–10 hpi of DC*avrRpm1* challenge^57^.

The *JISS1* reporter also responded to wounding stimuli, consistent with our previous microarray data^22^. The spatial expression pattern of JISS1–LUC activity mirrored jasmonate-dependent WASPs^40^. ETI-responding leaves triggered surface depolarizations (~100 mV) comparable to WASPs, but SISP generation and propagation dynamics were markedly slower. Conditional expression of *avrRpm1* in planta was insufficient to generate SISPs and required pathogen-associated molecular patterns (Fig. 5d and Extended Data Fig. 7d). Interestingly, wounding and herbivory release damage-associated molecular pattern molecules^59,60^. Our results suggest that PTI/ETI mutual potentiation^61^ may extend to SISP signalling.

Glutamate receptor-like mutants abolish WASPs^40,48^ and were required for SISP generation. They have previously been implicated in PTI^62^, resistance to pathogens^63^ and anti-herbivory defence^50,51^. *glr3.3a* exhibited markedly less depolarization of the challenged leaf (Fig. 6a, red trace) than *glr3.6a*. Notably, *jiss1* failed to induce SISPs; also, like *jiss1*, no tested *glr* mutants abolished ETI *JISS1*::*LUC* activity, and *glr3.3* *glr3.6* maintained SAR to *Psm4* (Fig. 6). Collectively, the loss of SISPs in *coi1-16* and *jiss1* (Figs. 5c and 6a) supports an additional role for jasmonates and JISS1 in SISP generation. SISPs may thus represent one of a suite of signals that collectively drive reprogramming of distal leaves to confer broad-spectrum resistance. Indeed, we previously showed that the generalist insect *Helicoverpa armigera* modified its feeding behaviour on *Arabidopsis* leaves, moving away from regions where *JISS1*::*LUC* was induced^64^.

Unlike JISS1, GLRs are believed to be ligand-gated channels. It remains unclear how either is activated to induce or maintain SISPs. More research is therefore necessary to understand the GLR mechanism of action in SISP generation. Localization of *JISS1*::JISS1–GFP predominantly to the epidermal ER and vasculature was unexpected. It is notable that both GLR3.3a and GLR3.6a localize to the leaf vasculature. Interestingly, GLR3.3a is primarily ER localized in phloem sieve elements, whereas GLR3.6a was predominantly in xylem contact cell tonoplast membranes^49,65^. Since distinguishing vascular cell types by cross-section is challenging, we examined single-cell transcriptomics data focusing on the vasculature^66^ and cell layers on the adaxial leaf surface^67^. The majority of *JISS1* vascular expression was in a discrete xylem parenchyma cluster, which also showed strong representation of genes encoding hormone pathways including JA as well as specific amino acid biosynthetic and degradation pathways^66^. *JISS1* expression was also evident in bundle sheath, adaxial procambium stem cells and a phloem parenchyma cluster, whereas epidermal and guard cell *JISS1* expression was seen in general cell-type clusters. Procko et al.^68^ identified *JISS1* in only 2 of 16 clusters corresponding to epidermal pavement and mesophyll/photosynthesis-related genes, both highly enriched for plant immune response transcripts.

In summary, *JISS1* encodes an ER-localized protein. The *JISS1* promoter functions as a dynamic spatial transcriptional SAR reporter, capturing propagative jasmonate-dependent signals rapidly moving through the vasculature, and symplastically via epidermal cells to systemic responding leaves. Our study reveals remarkable parallels between systemic wound signalling and the elicitation of SAR. Vascular jasmonate synthesis, such as in wounding, is well documented and has been attributed to electrical-signal-dependent remodelling of primary vein chloroplast galactolipids^65^. Here, *coi1*, *glr3.3a* *glr3.6a* and *jiss1* mutants all abolished SISPs, but the *glr* mutants (like *jiss1*) showed wild-type systemic JISS1–LUC signalling. This may represent an example of an amplification loop, considered widespread in systemic signalling^66^. Whereas ETI-mediated rapid jasmonate induction establishes defence against biotrophs, SISPs may propagate mobile information decoded systemically for anti-herbivory responses^50,51^. This study lays the foundation, and provides spatial–temporal tools, for dissecting the complex molecular mechanisms underpinning long-distance immune signalling.

## Methods

### *Arabidopsis* growth conditions

*Arabidopsis thaliana* were grown for four to five weeks in compost (Levingtons F2) in a controlled-environment growth chamber programmed at 60% relative humidity with 10 h days (21 °C; 120 μmol m^−2^ s^−1^) and 14 h nights (21 °C) as previously described^37,67^.

### Generating *Arabidopsis* transgenic lines

The *JISS1*::*LUC* plants were generated as previously described^64^. In brief, the *Photinus pyralis LUC2P* reporter gene containing the hPEST protein destabilization sequence (Promega pGL4.11) was cloned into pCAMBIA1302 via the Kpn1 and Pml1 restriction sites, creating pC1LUCP. A 1,631-bp promoter fragment of *JISS1* was PCR amplified from Col-0 genomic DNA and cloned into pC1LUCP using the primers detailed in Supplementary Table 2. Transgenic homozygous *JISS1*::*LUC* lines were generated in the Col-5 background via floral dipping^69^. Ten lines were screened via DC*avrRpm1* challenge, and the genomic location of *JISS1*::*LUC* in the strongest-expressing line was identified within At4g39240 using adapter ligation-based PCR^70^.

Subsequently, *JISS1*::*LUC* crosses into mutant plants were determined as homozygous via diagnostic PCR for *JISS1*::*LUC* insertion using the primers detailed in Supplementary Table 2. All other primers for validating mutants following crossing are shown in Supplementary Table 3. All homozygous *JISS1*::*LUC* mutant crosses were generated in the mutant parental Col-0 background.

The *JISS1*^*1–84*^*-GFP* line was generated using restriction enzyme cloning. The *JISS1* promoter and initial coding sequence were amplified from genomic DNA using the primers detailed in Supplementary Table 2. The PCR product was cloned into pCAMBIA1305 as a carboxy-terminal fusion with GFP. The *JISS1* promoter full-length *JISS1*::*GFP* line was generated using the Golden Gate assembly system^71^ as follows: the *JISS1* promoter was amplified as above and cloned into the level 0 plasmid pICH41295, while the full-length *JISS1* coding sequence was cloned into pAGM41287. The *JISS1* promoter and coding sequence were combined in the FL1P1 level 1 vector to create *JISS1*_*pro*_::*JISS1-GFP*::*Ocs*. Finally, the level 2 vector FL1P2 with BASTA selection was used to allow selection in planta after transformation into the Col-0 background via standard methods.

The homozygous *JISS1*_*pro*_::*JISS1-GFP* line was subsequently crossed with an *RFP-HDEL* (ER luminal marker) line (a gift from the late C. Hawes, Oxford Brookes University, UK).

The *JAZ10*::*GUS* line was a gift from E. Farmer (University of Lausanne), and the *npr1*, *npr1* *npr3* *npr4* and *nac19* *nac55* *nac72* mutants were a gift from X. Dong (Duke University). All other loss-of-function mutants and the Dex-*avrRpm1* line were obtained from the Nottingham *Arabidopsis* Stock Centre.

### Bacterial growth, maintenance and inoculation

Bacterial cultures (*P. syringae* pv. *tomato* strains DC containing the empty cloning vector (pVSP61), DC*hrpA* and DC containing the avirulence gene *avrRpm1*, *avrRps4* or *avrRpt2*) were grown in Kings B medium^72^ overnight with shaking (200 rpm) at 28 °C. Cells were harvested (2,000 *g* for 8 min), washed and resuspended in 10 mM MgCl_2_ (ref. ^67^). *avrRpm1* (in pVSP61) was introduced into the DC COR-deficient mutant DB4G3 (*cor*^−*1*^*/cor*^−*2*^)^38^. For luciferase, GUS, phenotyping and GFP assays, selected leaves were inoculated with a 1-ml needleless syringe on their abaxial surface with the appropriate bacterial suspension adjusted to a final optical density at 600 nm (OD_600_) of 0.15 in 10 mM MgCl_2_ (or as otherwise indicated in the figure legends).

For SAR growth assays, the immunizing inoculation comprised either 10 mM MgCl_2_ (mock) or OD_600_ 0.005 DC*avrRpm1*. Two days later, either *Psm4* or DC (see the figure legends) was inoculated at OD_600_ 0.001 or OD_600_ 0.002, respectively, using a similar protocol to ref. ^73^. Three days after DC or four days after *Psm4* challenges, bacterial growth measurements were determined from three inoculated leaves per plant and a minimum of four independent replicates. Significant growth differences between treatments were determined using Student’s *t*-test (unpaired two-tailed). All experiments were repeated at least three times.

### Chlorophyll fluorescence

Photosystem II chlorophyll fluorescence imaging of challenged leaves was performed with CF Imager software V2.305 (Technologica Ltd). Plants were placed in the chamber for 40 min post-inoculation and then dark adapted for 20 min. This was followed by a saturating light pulse (6,349 µmol m^−2^ s^−1^ for 0.8 s) to obtain maximum dark-adapted fluorescence (*F*_m_). Actinic light (120 µmol m^−2^s ^−1^) was then applied for 15 min, followed by a saturating pulse to obtain maximum light adapted fluorescence (*F*_m_′). The plants remained in actinic light for a further 24 min and were then returned to a dark period of 20 min. This cycle (59 min long) was repeated 23 times. *F*_m_, *F*_m_′ and *F*_o_ (minimal fluorescence with fully oxidized PSII centres) were used to calculate chlorophyll fluorescence parameters related to photosystem II: *F*_v_*/F*_m_ (maximum dark-adapted quantum efficiency) and non-photochemical quenching. Data were extracted using CF Imager software V2.305 (Technologica Ltd).

### Luciferase visualization

A solution of 1 mM luciferin (Promega) in 0.02% Silwet L77 (Loveland Industries, Ltd) was sprayed onto *JISS1*::*LUC* plants. The plants were kept in the dark for 30 min prior to inoculation. The petioles of the treated and adjacent rosette leaves were secured by folded paperclips to minimize epinastic movement. The plants were placed in a dark box, and images were captured using either an ORCAII ER CCD camera (Hamamatus Photonics) with a 35-mm f2.8 micro Nikkor lens or a Retiga R6 Scientific CCD camera (Qimaging) fitted with a Schneider STD XENON 25-mm lens. Photons were counted for 10 min at 2 × 2 binning mode, and data were acquired with either Wasabi (Hamamatsu) or Micro-Manager v.1.4 (Qimaging) software.

### Biophoton visualization

Pathogen-challenged plants were placed inside a dark box mounted with a Retiga R6 camera with a 25-mm f1.4 Navitar lens. Digital monochrome images captured photons for 20 min at 2 × 2 binning mode using MicroManager v.1.4. False-colouring, brightness adjusting and annotation were performed using Fiji (ImageJ2 v.2.9.0/1.53t)^74^, as described previously^20^.

### Chemicals

JA, SA, ABA, AZA, NA, Pip, DIECA, phenidone (1-phenyl-3-pyrazolidinone), lanthanum (III) chloride (LaCl_3_) and COR were all from Sigma. NHP was synthesized by Accel Pharmtech, and jarin-1 was sourced from AOBIOUS Inc. All chemicals were used at the concentrations described in the figures. DIECA (2.5 mM), phenidone (2 mM), jarin-1 (25 µM) or LaCl_3_ (1 mM) was pre-infiltrated into systemic leaves or co-infiltrated with DC*avrRpm1*, or the petiole of the challenged leaf was treated with the inhibitor soaked in cotton wool and secured in place with cling film.

### Wounding

Leaf tissue was wounded by crushing either side of the midrib with flat tweezers or by severing the petiole with scissors. Three leaves were wounded per plant, and three biological replicated were imaged.

### JAZ10–GUS expression

GUS activity in systemic leaves was assessed at 4, 8 and 24 hpi via GUS staining (1 mM X-Gluc, 100 mM NaPO_4_ buffer pH 7.0, 10 mM EDTA and 0.1% v/v Triton X-100) using four plants per treatment and challenging four leaves per plant. Samples were incubated at 37 °C. The leaves were de-stained by repeated washes with 70% ethanol, and representative leaf images are shown.

### Phenotyping

Infected leaves were removed and imaged at 5.5, 14 and 18 hpi to assess leaf collapse.

### Reverse transcription PCR

Total RNA was extracted from mature green leaves of *aos*, *jiss1* and Col-0 (two leaves per plant) using Trizol (Invitrogen, Thermo Fisher Scientific) and treated with DNase I (Merck), according to the manufacturers’ instructions. cDNA was synthesized using ReadyScript cDNA Synthesis Mix (Sigma-Aldrich) with both oligo d(T) and random primers and diluted 1:10 for PCR. PCR was performed using BioMix Red 2xPCR mix (Meridian Bioscience) with primers specific to *JISS1* (At5g56980; (LP) CAAGCATGTACGAAGCAAAT; (RP) CTCCTTGTACCTCAGAATCG; amplicon, 301 bp) or *Actin2* (At3g18780; (LP) GCCATCCAAGCTGTTCTCTC; (RP) CAGTAAGGTCACGTCCAGCA; amplicon, 156 bp).

### Confocal imaging

Freshly excised leaf samples were mounted in water and imaged on a Zeiss LSM 880 confocal microscope with a ×63 oil-immersion objective (ER images) or a ×10 air objective (whole-leaf images). GFP was excited at 488 nm, and emission was detected in the 498–559 nm range; RFP and chlorophyll A were excited at 561 nm and detected in the 605–649 nm range. For cell wall imaging, petiole sections were stained with an aqueous solution of propidium iodide (25 µM), with excitation at 561 nm and emission detection at 614–659 nm. All image analysis was performed in Fiji (ImageJ2 v.2.9.0/1.53t)^74^. Median pixel (signal) intensity for individual leaves was measured across the defined region of interest with corresponding background subtraction using Fiji.

### Electrophysiology experiments

Surface electrical potentials of challenged and systemic leaves were measured following infection using four electrodes (Fig. 5a) adopting a similar approach to ref. ^40^. The working electrode (W1, red) was always placed on the lamina immediately above the petiole of the challenged leaf. Electrodes W2 (green) and W3 (blue) were placed similarly on adjacent systemic leaves, and W4 (black) on the distal systemic leaf. The reference electrode was placed in the soil. Surface potential changes were measured from the reference electrode to the working electrode. Plants were covered with a propagator lid, electrical recordings were captured using a data logger (PicoLog 1000) and signal amplitude and duration were plotted for each leaf over ~24 h. Control recordings over an extended time predominantly showed a constant surface potential.

### Reporting summary

Further information on research design is available in the Nature Portfolio Reporting Summary linked to this article.

## Supplementary information

Supplementary Information Supplementary Tables 1–3.

Reporting Summary

Supplementary Video 1 Temporal spatial dynamics of luciferase activity in *JISS1::LUC* plants following DC*avrRpm1* (4:00 hpi; bottom left and top right plants), D*CavrRps4* (13:20 hpi; top left and top right plants) and DC*avrRpt2* (15:20 hpi; bottom right and top right plants). The white asterisk indicates infiltrated leaves. The video is false-coloured by signal intensity.

Supplementary Video 2 Temporal spatial dynamics of biophoton generation, indicative of chloroplast lipid peroxidation and associated with HR initiation, in wild-type Col-0 plants following challenge with D*CavrRpm1* (~3 hpi), DC*avrRpt2* (~13 hpi) or DC*avrRps4* (~16 hpi).

Supplementary Video 3 JISS^1–84^–GFP expression is predominantly restricted to the central vein and epidermal cell layer.

## Source data

Source Data Extended Data Figs. 4 and 5 Unprocessed reverse transcription PCR gels for Extended Data Figs. 4 and 5.

## Data Availability

The dataset for Extended Data Fig. 1 has been deposited at http://affymetrix.arabidopsis.info/narrays under identifier NASCARRAYS-403. The *A. thaliana* reporter lines and all raw data are available from the corresponding author. Source data are provided with this paper. All other data are available in the main text or supplementary materials.
