# Supplementary material for: Rapid local and systemic jasmonate signalling drives the initiation and establishment of plant systemic immunity
Source: Nat Plants. 2026 Jan 6;12(1):152–63. doi: 10.1038/s41477-025-02178-4 (PMC12830360; doi:10.1038/s41477-025-02178-4)
Supplement: Supplementary file 1 — Supplementary Tables 1–3. [file 41477_2025_2178_MOESM1_ESM.pdf]

nature plants

Supplementary information https://doi.org/10.1038/s41477-025-02178-4

Rapid local and systemic jasmonate signalling drives the initiation and establishment of plant systemic immunity

In the format provided by the authors and unedited

Supplementary Table 1: *JAZ* gene expression. Subsequently annotated *JAZ* genes significantly induced at 4 hpi with DC*avrRpm1* compared to DC/DC*hrpA* challenges, together with *JISS1* expression data in all treatments. Mean fold change ratios derived from normalised expression values on ATH1–121501 Affymetrix GeneChips as described[22]. The data set is deposited at http://affymetrix.arabidopsis.info/narrays under identifier NASCARRAYS-403.

Gene	Annotation	Mean fold change (DC*avrRpm1*/DC*hrpA*)
At1g19180	*JAZ1*	4.55
At1g74950	*JAZ2*	3.35
At3g17860	*JAZ3*	1.86
At1g17380	*JAZ5*	8.15
At1g72450	*JAZ6*	3.41
At2g34600	*JAZ7*	5.47
At1g30135	*JAZ8*	5.88
At1g70700	*JAZ9*	3.49
At5g13220	*JAZ10*	6.59
At1g48500	*JAZ4*	No probeset
At3g43440	*JAZ11*	No probeset
At5g20900	*JAZ12*	Not differentially expressed
At5g56980	*JISS1*	4.20

Supplementary Table 2: Primers used for generation of JISS1:LUC and JISS1:GFP lines. Primer purpose, forwards (F), reverse (R) and internal (I) primer name, and primer sequence given. Underlined nucleotides indicate recognition site for the corresponding restriction endonuclease where relevant.

Purpose of primers	Primer name	Primer sequence
Cloning JISS1:LUC	JISS1F + KpnI	AATCCATGGTCAACCGTAAAAGGTCGGTGTAG
	JISS1R + NcoI	AACTCCATGGTTGGGTTGTGTTTTATGTTGGTTTTG
Confirming JISS1:LUC insertion	JISS1:LUC T-DNA F	GTCCTTGGTGGATGCATTGAT
	JISS1:LUC T-DNA R	CTCCGTGCAACAGATTTTGGTT
	JISS1:LUC T-DNA I	GATCCCCCGAATTAATTCGGCG
Cloning JISS1:GFP	JISS1:GFP pro F	TCAAAGAATTCCGTAAAAGGTCGGTGTAGC
	JISS1:GFP R	GTGCCATGGAGAAAAGCTCAGTTTCTGGATG

Supplementary Table 3: Primers for identification of homozygous T-DNA lines after crosses. Primers for the *npr1/3/4* triple mutant which were obtained from Xinnian Dong (Duke University)[28]. All primers used for genotyping were designed by the Salk Institute Genomic Analysis Laboratory.

**Mutant**	**Gene**	**t- DNA**	**Primers Sequence (5' to 3')**	**KO. Product size**
sid2	AT1G74710	SALK_133146.39.30	LP - TCTGATGGATCTCCAATCGTC<br>RP - GAGATTTCAAGACGCCACTTG	577-877
nac055	AT3G15500	SALK_014331.54	LP - TAAACGATGAGCGATAGCGAG<br>RP - AAAGGAACCAAAACCAATTGG	467-767
nac019	AT1G52890	SALK_096295.49.30	LP - TCAATGAACTCAAGGGATTGC<br>RP - ATGCGGTTTGGGTTAGAAAAC	459-759
nac072	AT4G27410.2	SALK_083756.50.50	LP - GACTGGTCTTTTATCTCCGGG<br>RP - ACAACACATCGATAAGGTCGG	527-827
jiss1	AT5G56980	SALK_002838.49.30	LP-ATGTTTACCCGGATCCAAATC<br>RP-GCCACACATACTTCGCTAAGC	552-852
coi1-16	AT2G39940	SALK_045434		
fmo1	AT1G19250	SALK_026163	LP- CTTTTCGGTTGGACTTGGAAC<br>RP- CTGCTTTGGACGTATCCTACG	485-785
aos	AT5G42650	SALK_017756	LP- CGAGAAATTAACGGAGCTTCC<br>RP-CTAACCGGAGGCTACCGTATC	432-732
glr3.3a	AT1G42540	SALK-099757	LP-GATGCTGCATATGGTTGTGTG<br>RP-GTTGAACGATAAGCTTGCGAG	700
glr3.6a	AT3G51480	SALK_091801	LP-TTCGTTCAAAGGTGGCATAAC<br>RP-CGACTATGAGGAAAGACGCAG	550
npr3* (deleted)	AT5G45110	SALK_043055	LP1-TGATTGTTGTCGACCTGCCA<br>RP1-AGATCTGACCTCGCCACTCT<br>LP2-<br>TTGGTTCTTTTGCCTTCTCTTTGA<br>RP2-GGCATCCCTATCACCATCTGT	307<br><br>209
npr4* **(deleted)**	AT4G19650	SALK_098460	LP1-TTGGCGATGAAGCTAAGGGG<br>RP1-CTGGCAGAGAGCATGAACCA	526

			LP2- TACGCTACTGCTGTTCCAGA<br>RP2-CTTGCACGTGTGCTTTTTGG	341
npr1*	AT1G64280	EMS mutagenized	LP-CTCGAATGTACATAAGGCAC<br>RP-GTGCGGTTCTACCTTCC	296
