# Supplementary material for: Rapid local and systemic jasmonate signalling drives the initiation and establishment of plant systemic immunity
Source: Nat Plants. 2026 Jan 6;12(1):152–63. doi: 10.1038/s41477-025-02178-4 (PMC12830360; doi:10.1038/s41477-025-02178-4)
Supplement: Supplementary file 2 — Reporting Summary [file 41477_2025_2178_MOESM2_ESM.pdf]

nature portfolio

Corresponding author(s): Murray Grant
Last updated by author(s): 7th November 2025

Reporting Summary

Nature Portfolio wishes to improve the reproducibility of the work that we publish. This form provides structure for consistency and transparency in reporting. For further information on Nature Portfolio policies, see our Editorial Policies and the Editorial Policy Checklist.

Statistics

For all statistical analyses, confirm that the following items are present in the figure legend, table legend, main text, or Methods section.

n/a	Confirmed	
☐	☒	The exact sample size (*n*) for each experimental group/condition, given as a discrete number and unit of measurement
☒	☐	A statement on whether measurements were taken from distinct samples or whether the same sample was measured repeatedly
☐	☒	The statistical test(s) used AND whether they are one- or two-sided<br>*Only common tests should be described solely by name; describe more complex techniques in the Methods section.*
☒	☐	A description of all covariates tested
☒	☐	A description of any assumptions or corrections, such as tests of normality and adjustment for multiple comparisons
☒	☐	A full description of the statistical parameters including central tendency (e.g. means) or other basic estimates (e.g. regression coefficient) AND variation (e.g. standard deviation) or associated estimates of uncertainty (e.g. confidence intervals)
☐	☒	For null hypothesis testing, the test statistic (e.g. *F*, *t*, *r*) with confidence intervals, effect sizes, degrees of freedom and *P* value noted<br>*Give P values as exact values whenever suitable.*
☒	☐	For Bayesian analysis, information on the choice of priors and Markov chain Monte Carlo settings
☒	☐	For hierarchical and complex designs, identification of the appropriate level for tests and full reporting of outcomes
☒	☐	Estimates of effect sizes (e.g. Cohen's *d*, Pearson's *r*), indicating how they were calculated

Our web collection on statistics for biologists contains articles on many of the points above.

Software and code

Policy information about availability of computer code

Data collection	Chlorophyll fluorescence measurements were carried out using a CF Imager (Technologica Ltd, Colchester, UK) and data extracted and analysed using the FluorImager software V2.305. Luciferase visualisation and biophoton data acquisition was performed using Micro-Manager 1.4 (QImaging)
Data analysis	Image analysis (confocal and biophotons) was performed using Fiji (ImageJ2 version 2.9.0/1.53t)

For manuscripts utilizing custom algorithms or software that are central to the research but not yet described in published literature, software must be made available to editors and reviewers. We strongly encourage code deposition in a community repository (e.g. GitHub). See the Nature Portfolio guidelines for submitting code & software for further information.

Data

Policy information about availability of data

All manuscripts must include a data availability statement. This statement should provide the following information, where applicable:

- Accession codes, unique identifiers, or web links for publicly available datasets
- A description of any restrictions on data availability
- For clinical datasets or third party data, please ensure that the statement adheres to our policy

All data is available in the main text or the supplementary materials. Arabidopsis thaliana reporter lines are available from the corresponding author.

Research involving human participants, their data, or biological material

Policy information about studies with human participants or human data. See also policy information about sex, gender (identity/presentation), and sexual orientation and race, ethnicity and racism.

Reporting on sex and gender	N/A
Reporting on race, ethnicity, or other socially relevant groupings	N/A
Population characteristics	N/A
Recruitment	N/A
Ethics oversight	N/A

Note that full information on the approval of the study protocol must also be provided in the manuscript.

Field-specific reporting

Please select the one below that is the best fit for your research. If you are not sure, read the appropriate sections before making your selection.

☒ Life sciences ☐ Behavioural & social sciences ☐ Ecological, evolutionary & environmental sciences

For a reference copy of the document with all sections, see nature.com/documents/nr-reporting-summary-flat.pdf

Life sciences study design

All studies must disclose on these points even when the disclosure is negative.

Sample size	Chlorophyll fluorescence, biophotons, and luciferase imaging:<br>Sample size per experiment is limited to 4 plants due to the size constraints of the imaging cabinet. Each experiment was performed at least 3 times (and often in excess of 10 times) with comparable results.<br><br>Confocal microscopy:<br>JISS1:JISS1-GFP lines were imaged on several occasions with multiple epidermal cells viewed each time and images captured from n=8-10 representative cells/experiment.<br><br>Electrophysiology:<br>Due to the nature of the experimental set up it is only possible to capture measurements from one plant per experiment, hence the data was obtained from n=1. However, each of the presented experiments was repeated multiple times with comparable traces produced, as shown in the Extended Data.<br><br>SAR growth assays:<br>Each SAR experiment used 6 plants per mutant as determined by practical experimental constraints. Each experiment was repeated at least 3 times, and the SAR experiments on the jiss1 mutant were completed more than 10 times in two independent laboratories using similar protocols (detailed in methodology).<br><br>JAZ10:GUS expression: For each bacterial treatment (DChrpA, DCavrRpm1 or DC) four leaves on four plants (JAZ10:GUS Col-0 or JAZ10:GUS coi1-16) were challenged. At each timepoint (4h, 6h, 8h) one unchallenged (systemic) leaf was harvested from each plant and assayed for GUS activity. Two representative images from the four assayed leaves are shown in Fig 3A.<br><br>RT-PCR: RNA was extracted from leaf material from n=3-4 plants (2 leaves per plant) with 3 independent batches of plants grown in total ie n=9-12 (Note: this was part of a larger experiment where some plants were infected with DC3000, hence the high level of replication)
Data exclusions	SAR assays are known to be highly variable (Rufian et al., 2019). Each SAR experiment was performed on n=6 plant/treatment/mutant. We pre-established exclusion criteria such that data would be excluded from 1. any plant exhibiting secondary (opportunistic) fungal infection 2. any plant exhibiting bacterial counts at least 1 log fold base 10 different to the median of the remaining plants with a maximum of 2 plants excluded from each mutant/treatment group (ie minimum of n=4); if n<4 following this exclusion criteria then the whole experiment was discarded and repeated.
Replication	Biophoton experiments are variable in terms of the exact timings when the signal is observed (typically within a 2 hour window) but also in that some infiltrated leaves do not produce a detectable biophoton signal. On occasion it was therefore not possible to observe detectable signals from all three bacterial challenges on the same plant in order to produce a single figure panel (as presented in Extended Data Fig. 2C). However, we consistently saw leaves producing a biophoton signal in the temporal order presented ie DCavrRpm1, then DCavrRpt2 then DCavrRps4. The exact timing of signal generation was also highly consistent between plants in the same experiment.
Randomization	Trays of 24 Arabidopsis plants/line are grown under controlled conditions for 5-6 weeks prior to experimentation. Plants within a tray are thus highly comparable in terms of growth and development. Multiple plants from the same tray are selected for an experiment and randomly

	assigned to a treatment group. Where multiple lines are used in an experiment all plants were sown on the same day and grown under the same conditions thus ensuring inter-line consistency in growth and development.
Blinding	Blinding was not performed due to researcher personnel constraints and the need to ensure adequate sample labelling and tracking.

Reporting for specific materials, systems and methods

We require information from authors about some types of materials, experimental systems and methods used in many studies. Here, indicate whether each material, system or method listed is relevant to your study. If you are not sure if a list item applies to your research, read the appropriate section before selecting a response.

Materials & experimental systems

n/a	Involved in the study	
☒	☐	Antibodies
☒	☐	Eukaryotic cell lines
☒	☐	Palaeontology and archaeology
☐	☒	Animals and other organisms
☒	☐	Clinical data
☒	☐	Dual use research of concern
☐	☒	Plants

Methods

n/a	Involved in the study	
☒	☐	ChIP-seq
☒	☐	Flow cytometry
☒	☐	MRI-based neuroimaging

Animals and other research organisms

Policy information about studies involving animals; ARRIVE guidelines recommended for reporting animal research, and Sex and Gender in Research

Laboratory animals	N/A
Wild animals	N/A
Reporting on sex	N/A
Field-collected samples	N/A
Ethics oversight	N/A

Note that full information on the approval of the study protocol must also be provided in the manuscript.

Dual use research of concern

Policy information about dual use research of concern

Hazards

Could the accidental, deliberate or reckless misuse of agents or technologies generated in the work, or the application of information presented in the manuscript, pose a threat to:

No	Yes	
☒	☐	Public health
☒	☐	National security
☐	☒	Crops and/or livestock
☐	☒	Ecosystems
☒	☐	Any other significant area

Hazards	This work involved the generation and growth of GM plants and notably the plant pathogen, Pseudomonas syringae pv. tomato, the latter to alter the effector protein repertoires. All work was carried out under the necessary levels of oversight, authorisation and risk management mandated by the host University and subject to UK legislation. There is no evidence to suggest that the GM plants generated could compete with and/or displace other plants, be more toxic to animals, cause harm to beneficial microorganisms or exhibit altered altered interactions with plant pathogens with adverse effects relative to the equivalent unmodifed plant. Similarly, the genetic modification of Pseudomonas syringae will not alter the ability of any escaping GMO to cause disease in humans.

For examples of agents subject to oversight, see the United States Government Policy for Institutional Oversight of Life Sciences Dual Use Research of Concern.

Experiments of concern

Does the work involve any of these experiments of concern:

No	Yes	
☒	☐	Demonstrate how to render a vaccine ineffective
☒	☐	Confer resistance to therapeutically useful antibiotics or antiviral agents
☒	☐	Enhance the virulence of a pathogen or render a nonpathogen virulent
☒	☐	Increase transmissibility of a pathogen
☒	☐	Alter the host range of a pathogen
☒	☐	Enable evasion of diagnostic/detection modalities
☒	☐	Enable the weaponization of a biological agent or toxin
☒	☐	Any other potentially harmful combination of experiments and agents

Precautions and benefits

Biosecurity precautions	All research undertaken using GMOs is subject to the necessary levels of oversight, authorisation and risk management as prescribed by our University's Genetic Modification and Biosafety Committee and by current UK legislation. Consequently, comprehensive risk assessments are in place which detail potential hazards, inserted genetic material, vectors and risks posed to the environment alongside appropriate control and containment measures, disposal and emergency procedures.<br>Specifically to the use of Pseusomonas syringae DC3000, a non-native UK phytopathogen, the corresponding author Prof Murray Grant, holds a DEFRA license for the use of this pathogen for research purposes and abides by DEFRA-mandated practises to mitigate against accidental or deliberate release.
Biosecurity oversight	See above
Benefits	An increased understanding of plant-pathogen interactions will aid in the development of crops that are more resistent to plant pathogens and help mitigate against the significant current losses in yield both pre- and post-harvest thus impacting future global food security.
Communication benefits	We do not foresee how any harm could result from the publication of this work and therefore publication can only be beneficial (or neutral) to both academics and the wider public.

Plants

Seed stocks	Arabidopsis thaliana reporter lines are available from the corresponding author
Novel plant genotypes	Detailed in Methods section/Origin of transgenic Arabidopsis lines
Authentication	Detailed in Methods section/Origin of transgenic Arabidopsis lines
