# Supplementary material for: Rapid local and systemic jasmonate signalling drives the initiation and establishment of plant systemic immunity
Source: Nat Plants. 2026 Jan 6;12(1):152–63. doi: 10.1038/s41477-025-02178-4 (PMC12830360; doi:10.1038/s41477-025-02178-4)
Supplement: Supplementary file 6 — Unprocessed reverse transcription PCR gels for Extended Data Figs. 4 and 5. [file 41477_2025_2178_MOESM6_ESM.pdf]

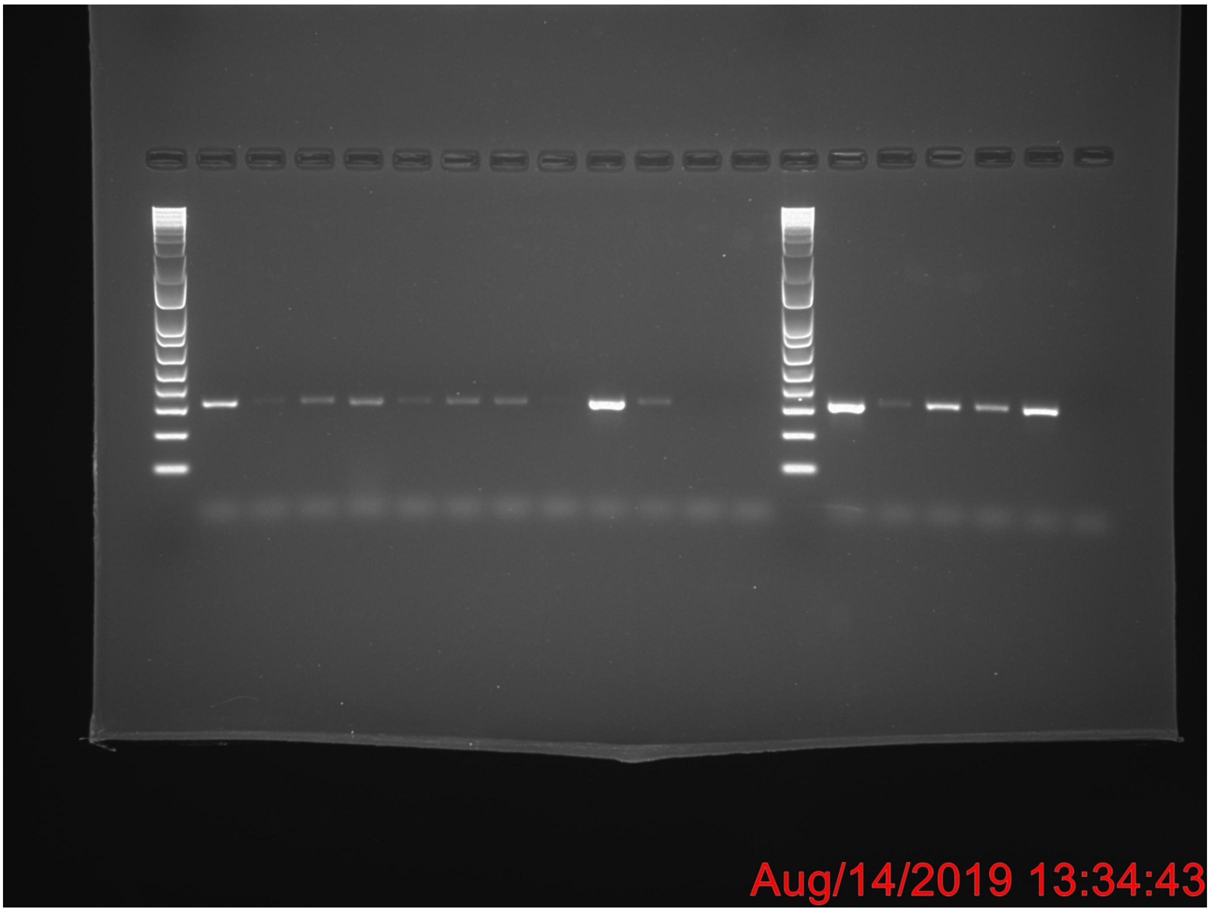

Unprocessed RT-PCR gel showing *JISS1* expression in Col-0 and *aos*. Wells 14 – 16 are presented in Extended Data Fig. 4A (left).

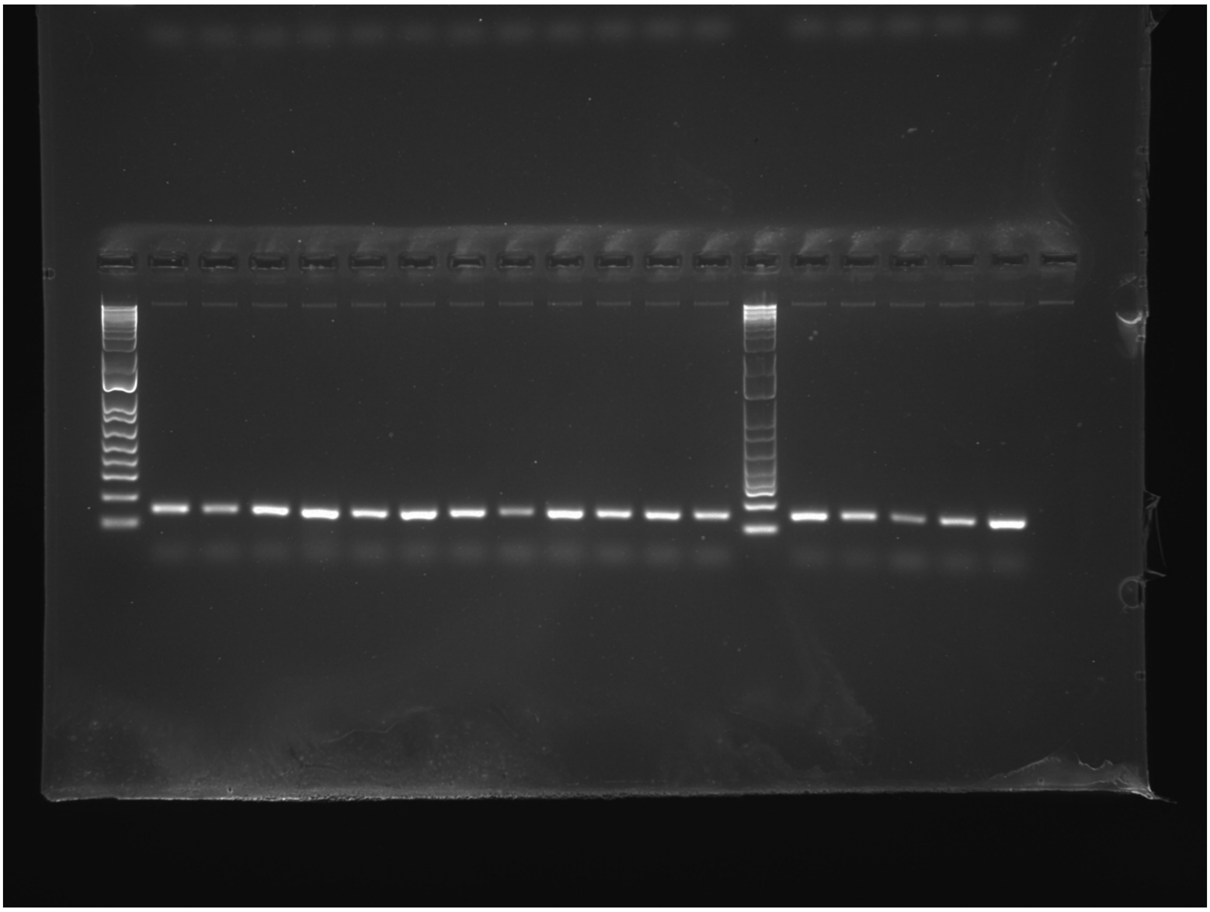

Unprocessed RT-PCR gel showing *Actin2* expression in Col-0 and *aos*. Wells 14 – 16 are presented in Extended Data Fig. 4A (right).

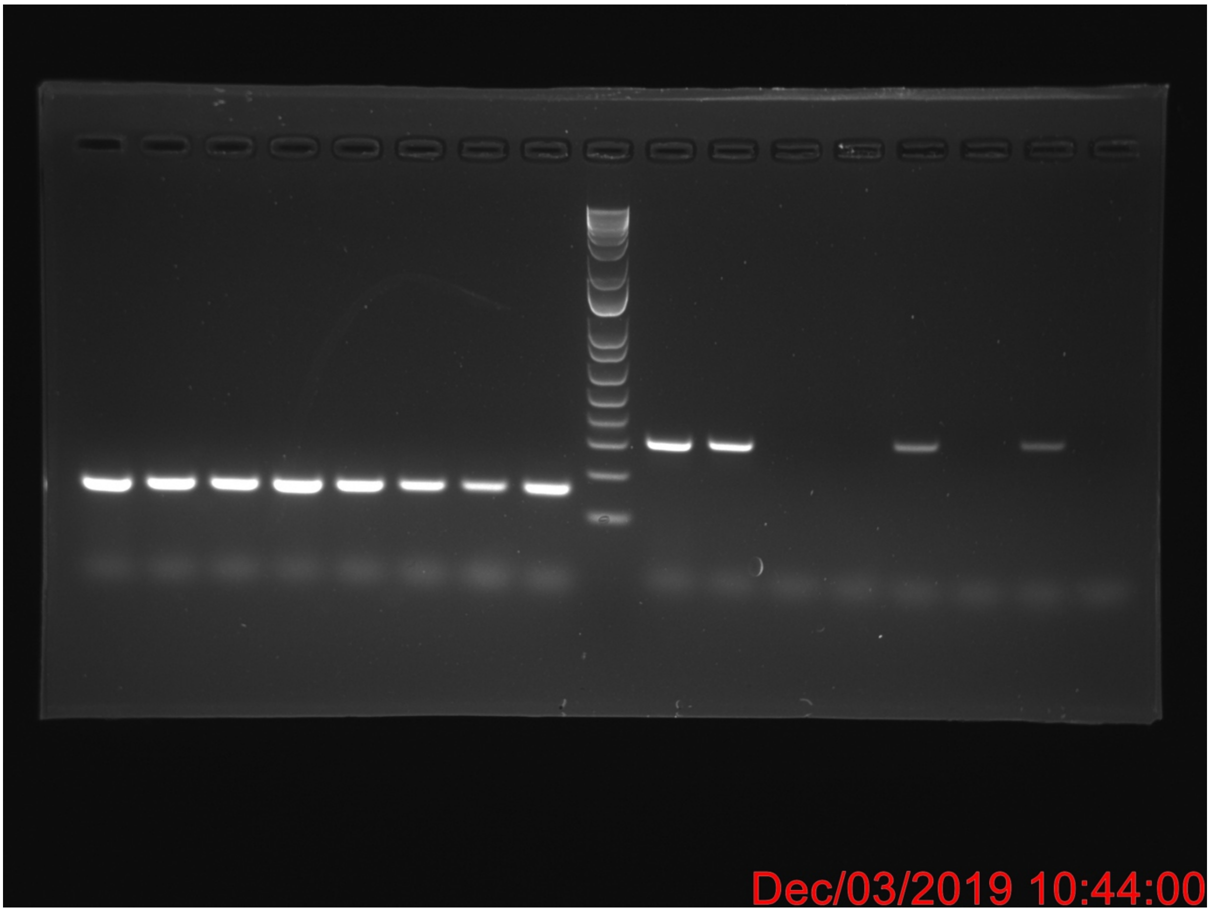

Unprocessed RT-PCR gel showing *Actin2* (left of ladder) and *JISS1* (right of ladder) expression in Col-0 and *jiss1*. Wells 5 - 13 are presented in Extended Data Fig. 5A.
